# Evaluation of the Diagnostic Capability of Spectralis SD-OCT 8 × 8 Posterior Pole Software with the Grid Tilted at 7 Degrees and Horizontalized in Glaucoma

**DOI:** 10.3390/jcm13041016

**Published:** 2024-02-09

**Authors:** Aurora Alvarez-Sarrion, Jose Javier Garcia-Medina, Ana Palazon-Cabanes, Maria Dolores Pinazo-Duran, Monica Del-Rio-Vellosillo

**Affiliations:** 1 Department of Ophthalmology and Optometry, University of Murcia, 30120 Murcia, Spain; aurora.alvarezopt@gmail.com; 2 Department of Ophthalmology, General University Hospital Morales Meseguer, 30008 Murcia, Spain; 3 Ophthalmic Research Unit “Santiago Grisolia”, 28029 Valencia, Spain; dolores.pinazo@uv.es; 4 Spanish Net of Inflammatory Diseases RICORS, Institute of Health Carlos III, 28029 Madrid, Spain; 5 Department of Ophthalmology, Hospital Virgen del Castillo, Yecla, 30510 Murcia, Spain; a.palazoncabanes@gmail.com; 6 Cellular and Molecular Ophthalmo-Biology Group, Department of Surgery, University of Valencia, 46010 Valencia, Spain; 7 Department of Anesthesiology, General University Hospital Morales Meseguer, 30008 Murcia, Spain; monica.delrio@um.es; 8 Department of Surgery, Obstetrics and Gynecology and Pediatrics, University of Murcia, 30120 Murcia, Spain

**Keywords:** glaucoma, posterior pole, macula, thickness, layer, segmentation, map, 8 × 8, sensitivity, specificity

## Abstract

**Background**: The goal was to evaluate the diagnostic capability of different parameters obtained with the posterior pole (PP) software in Spectralis SD-OCT with the 8 × 8 grid tilted at 7° and horizontalized in glaucomatous eyes. **Methods**: A total of 299 eyes were included, comprising 136 healthy eyes and 163 with primary open-angle glaucoma (POAG). The following segmentations were evaluated: complete retina, retinal nerve fiber layer (RNFL), ganglion cell layer (GCL), GCL and inner plexiform layer (GCLIPL), ganglion cell complex (GCC), outer plexiform layer and outer nuclear layer (OPLONL), inner retinal layer (IRL), and outer retinal layer (ORL). Different patterns of macular damage were represented using heatmaps for each studied layer, where the areas under the curve (AUROC) values and a retinal thickness cutoff point were defined to discriminate POAG patients. Results: There was not any difference in the diagnostic capability for detecting glaucoma between the grid tilted at 7° and horizontalized. The macular segmentations that offer the highest diagnostic ability in glaucoma discrimination were, in the following order, RNFL (AUROC = 0.796), GCC (AUROC = 0.785), GCL (AUROC = 0.784), GCLIPL (AUROC = 0.770), IRL (AUROC = 0.755), and the complete retina (AUROC = 0.752). In contrast, ORL and OPLONL do not appear to be helpful for discriminating POAG. **Conclusions**: Some results of PP software may be useful for discriminating POAG.

## 1. Introduction

Primary open-angle glaucoma (POAG), in most cases, begins as a disease of the anterior segment of the eye, where there is increased resistance to the drainage of aqueous humor, resulting in elevated intraocular pressure (IOP), or pressure that is too high for the patient’s eye [1]. This condition causes a progressive and chronic optic neuropathy [2,3] characterized by optic nerve (ON) atrophy, papillary excavation, increased IOP, and a decrease in intraretinal thickness [4,5,6]. It has been classified as one of the leading causes of blindness worldwide [3,7,8].

An emerging method for diagnosing POAG and monitoring its progression is through the use of ON imaging and measurements of different retinal thicknesses with optical coherence tomography (OCT) [9,10,11]. Scientific evidence of early and even initial glaucomatous damage in the macula can be found in the literature. The assessment of macular thickness can be a valuable tool in evaluating glaucomatous structural changes [12,13,14,15,16]. To detect this damage with OCT, a macular scan should be obtained [17]. Additionally, understanding sequences of macular damage and specific macular patterns can provide relevant information for monitoring glaucomatous progression [18].

OCT allows for the quantitative evaluation of structural parameters of the retina [19,20,21]. The posterior pole (PP) software in Spectralis SD-OCT (Heidelberg Engineering, Germany) provides the total value of retinal thickness in an automatically tilted 8 × 8 grid (with 64 macular cells or superpixels) aligned at 7 degrees, which is the more common disc-fovea axis inclination. Other devices and software do not take this inclination into account and present the different grids horizontalized.

Furthermore, Spectralis SD-OCT allows for the segmentation of different retinal layers to obtain the isolated thickness of each layer or the thickness resulting from the combination of different layers. It is unknown which method offers the best results. Therefore, the objective of this study is to evaluate the diagnostic capacity of the PP algorithm in Spectralis SD-OCT with the 8 × 8 grid, tilted at 7° and horizontally aligned in glaucomatous eyes, through the analysis of the area under the curve (AUROC) values of thickness patterns obtained from the segmentation of different retinal layers or sets of layers with both inclinations.

## 2. Materials and Methods

**Figure 1:**
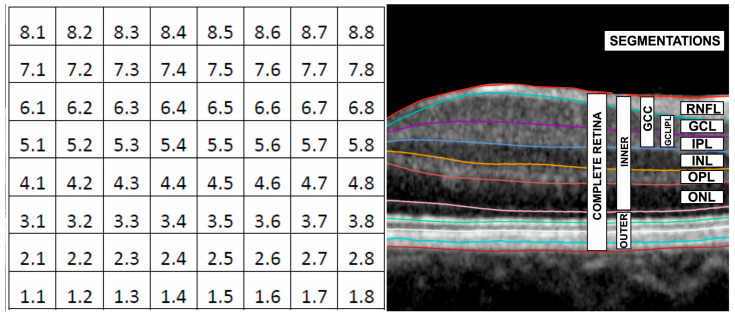
Denomination of cells or superpixels in the 8 × 8 posterior pole algorithm. The cells are named 1.1 at the lower temporal corner, 1.8 at the lower nasal corner, 8.1 at the upper temporal corner, and 8.8 at the upper nasal corner (**left figure**). Spectralis SD-OCT segmentations are also shown (**right figure**). RNFL = retinal nerve fiber layer, GCL = ganglion cell layer, IPL = inner plexiform layer, INL = inner nuclear layer, OPL = outer plexiform layer, ONL = outer nuclear layer, GCC = ganglion cell complex, GCLIPL = GCL+IPL, INNER = inner retina layer (IRL), OUTER = outer retina layer (ORL).

This is a cross-sectional study that includes a total of 299 eyes, comprising 136 healthy eyes and 163 eyes with POAG. The Spectralis SD-OCT PP 8 × 8 software was employed for the study. This algorithm is made up of 64 cells or superpixels. The following segmentations were evaluated: complete retina, retinal nerve fiber layer (RNFL), ganglion cell layer (GCL), GCL and inner plexiform layer (GCLIPL), ganglion cell complex (RNFL+ GCLIPL), combination of outer plexiform layer and outer nuclear layer (OPLONL), inner retinal layers (IRL), and outer retinal layers (ORL) (Figure 1).

Inclusion criteria were defined as follows: obtaining PP maps centered on the fovea, correctly segmented OCT scans, patients diagnosed solely with POAG (case group), healthy subjects (control group), and subjects of Caucasian race. Exclusion criteria encompassed decentered PP maps from the fovea, poorly segmented OCT scans or inadequate image quality (signal strength < 20), individuals with ocular pathologies other than POAG or general pathologies that could affect macular thickness determination, best-corrected visual acuity (BCVA) less than 20/60, and individuals with isolated ocular hypertension.

**Figure 2:**
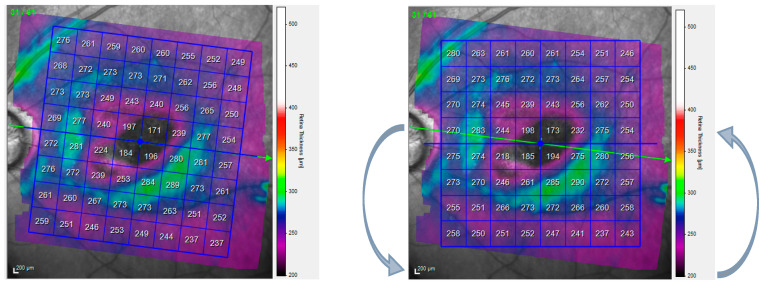
8 × 8 grid of the posterior pole (PP) from Spectralis SD-OCT automatically tilted 7 degrees by the device (**left**). Horizontalized 8 × 8 grid of PP from Spectralis SD-OCT (**right**).

Regarding the data collection method, the research was conducted at the University General Hospital Reina Sofía (Murcia, Spain), with 632 eyes classified as healthy or glaucomatous. The study protocol adhered to the ethical principles of the Declaration of Helsinki and was approved by the Local Ethics Committee at the University General Hospital Reina Sofía in Murcia, Spain (protocol number 02/18). Initially, demographic and ophthalmological characteristics of both groups (gender, age, IOP), optic disc cupping, mean deviation (MD), pattern standard deviation (PSD), and the presence of other pathologies were compiled (results in Table 1). Subsequently, retinal thickness data collection occurred in two phases. First, eyes were identified in Spectralis SD-OCT, and PP maps were selected, automatically tilted by 7 degrees, checked for appropriate segmentation, and thickness values from different segmentations were exported. Then, manual horizontalization of the PP 8 × 8 grid was performed (Figure 2), and thickness values were exported again. Each cell was symmetrically labeled according to whether it was the right or left eye. All eyes were considered and depicted as if they were right eyes in this study.

### Statistical Analysis

The statistical analysis was conducted using SPSS 27.0 for Windows. Differences were considered statistically significant when *p* < 0.05. 

A descriptive analysis of qualitative and quantitative variables was performed, along with a comparison of means for both quantitative and qualitative variables. The diagnostic capability of the thickness of each cell in the 8 × 8 grid was calculated and represented in heatmaps. Subsequently, two global indices, mean and weighted, were calculated. Global indices were compared between grids and between layers within the same grid. Finally, cutoff points were determined for each layer and diagnostic capability indices. 

In the descriptive analysis of qualitative variables, the number of cases in each category and their corresponding percentages were obtained, while for quantitative variables, minimum and maximum values, means, and standard deviation were calculated. 

For the comparison of means of quantitative variables between the two groups, the independent samples t-test was employed after verifying the assumptions of normality with the Kolmogorov–Smirnov test. For qualitative variables, comparisons between the groups were performed using the Pearson chi-squared test. 

The diagnostic capability of retinal macular thickness was calculated using the area under the curve (AUROC) for each cell in the 8 × 8 grid in each layer and in both grids. This was represented in heatmaps, where AUROC ≤ 0.5 was shown in blue, between 0.6 and 0.69 in white, and AUROC ≥ 0.70 in red. 

To calculate the global indices for each layer or combination of layers studied in both grids, cells with AUROC ≥ 0.70 were selected. Two global indices were calculated—mean and weighted indices—and were compared using a related samples design. The mean index is the average thickness of selected cells, while the weighted index is the average of thickness values multiplied by the AUROC of the selected cells. These indices were compared in each segmentation and in each grid with the DeLong test. 

Finally, cutoff points of the mean index in the inclined grid were determined for each layer, establishing the maximum thickness to classify a patient as diseased. Diagnostic validity indices were then calculated with 95% confidence intervals: sensitivity, specificity, positive predictive value (PPV), and negative predictive value (NPV). 

## 3. Results

### 3.1. Analysis of Demographic and Ophthalmic Data

The final sample of the study consisted of 299 eyes from 185 subjects, with 47.6% being male (*n* = 88) and 52.4% female *(n* = 97). The mean age of the subjects was 71.6 years (Min.–Max.: 20–97, SD = 14.9). The number of eyes and subjects participating in the study were compared between healthy and glaucomatous groups based on gender, age, BCVA, IOP, optic disc excavation, mean deviation (MD), and pattern standard deviation (PSD). Significant differences were found between the two groups in optic disc excavation, MD, PSD, and age, with healthy subjects being younger than diseased subjects (Table 1). 

### 3.2. Analysis of AUROCs

Supplementary Tables S2–S8 present the AUROCs, 95% confidence intervals, and *p*-values for the layers and layer combinations studied with the 7° inclined and horizontalized PP grids. 

**Figure 3:**
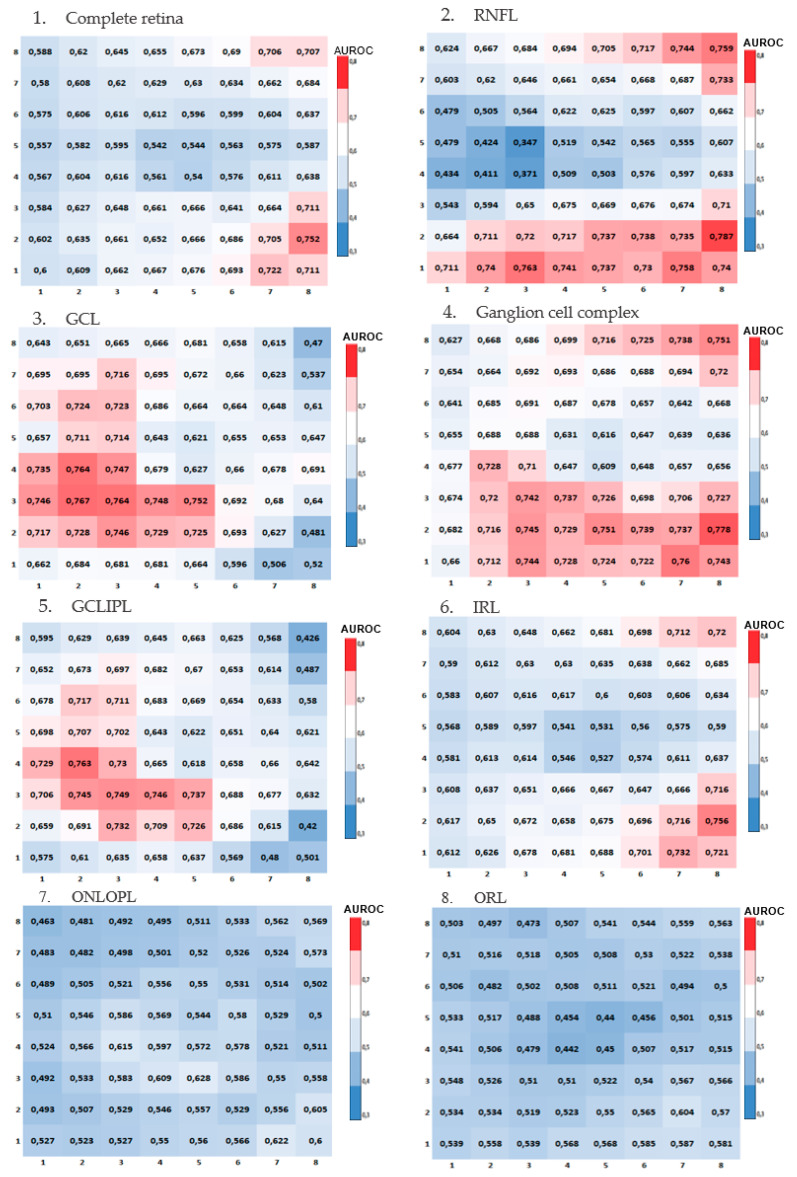
Heatmaps of AUROCs for the studied layers in the horizontalized grid using the 8 × 8 grid of the posterior pole from Spectralis SD-OCT. (**1**) Complete retina. (**2**) Retinal nerve fiber layer (RNFL). (**3**) Ganglion cell layer (GCL). (**4**) Ganglion cell complex. (**5**) Ganglion cell layer + inner plexiform layer (GCLIPL). (**6**) Inner retinal layer (IRL). (**7**) Outer nuclear layer + outer plexiform layer (ONLOPL). (**8**) Outer retinal layer (ORL).

**Figure 4:**
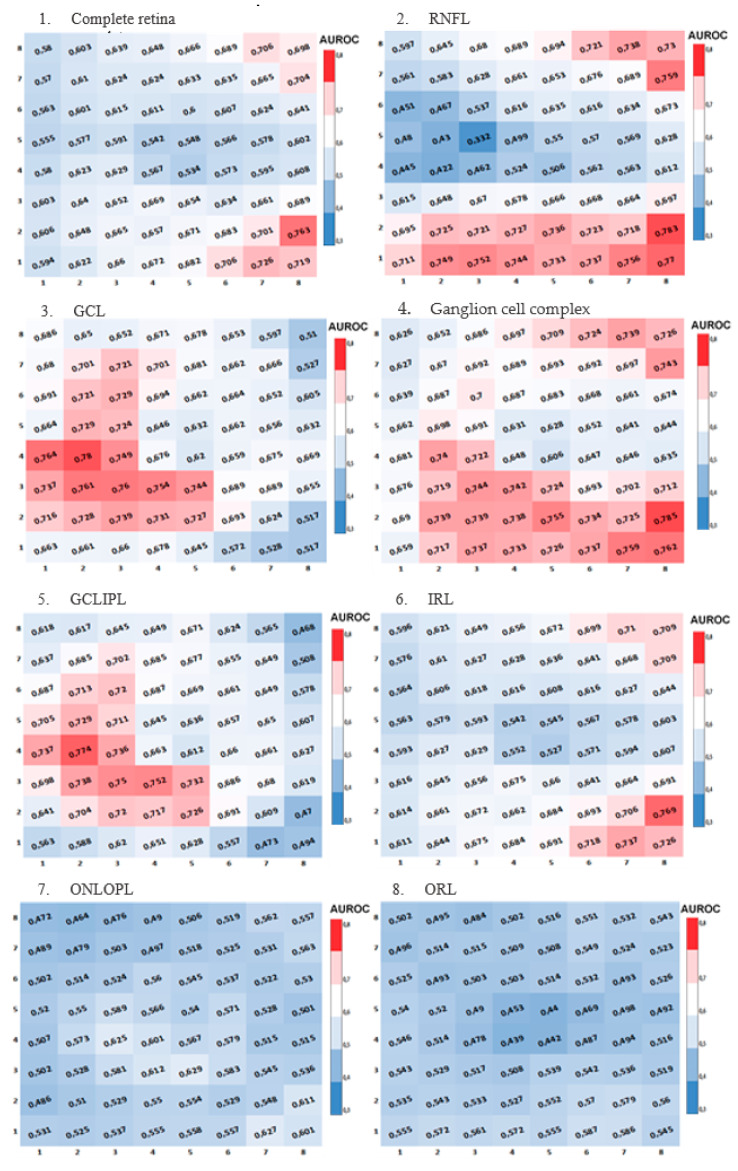
Heatmaps of AUROCs for the studied layers in the inclined grid (disc-fovea axis) using the 8 × 8 grid of the posterior pole from Spectralis SD-OCT. (**1**) Complete retina. (**2**) Retinal nerve fiber layer (RNFL). (**3**) Ganglion cell layer (GCL). (**4**) Ganglion cell complex. (**5**) Ganglion cell layer + inner plexiform layer (GCLIPL). (**6**) Inner retinal layer (IRL). (**7**) Outer nuclear layer + outer plexiform layer (ONLOPL). (**8**) Outer retinal layer (ORL).

The heatmaps of AUROC for each studied layer (RNFL, GCL) and layer combinations (complete retina, IRL, ORL, GCLIPL, OPLONL, and ganglion cell complex) are then presented for each cell in the horizontalized (Figure 3) and 7° inclined (Figure 4) grids. Blue cells indicate lower diagnostic capability. Red cells represent a greater AUROC, and consequently, a higher diagnostic capability and more significant changes in retinal thickness between healthy and glaucomatous subjects. 

Across all the heatmaps, in all studied layers and layer combinations, a very similar pattern is observed between the results obtained with the inclined and horizontalized 8 × 8 grids. The ganglion cell complex is the segmentation that shows the most cells with good diagnostic capability (AUROC ≥ 0.7). 

### 3.3. Analysis of Global Indices

Following the calculation of AUROC for each cell in the 8 × 8 PP grid in the studied layers, both in the 7° inclined and horizontal grids, two global indices were calculated: mean index and weighted index. The AUROC obtained for these indices did not show statistically significant differences in any of the layers and grids (*p* > 0.05, DeLong test). Therefore, the mean index was chosen to continue the research, as it is a more straightforward methodology for replication in any future investigation. Supplementary Tables S9–S14 show the AUROC for each layer and comparisons between indices based on the grid position. The AUROC for the global indices of the ONLOPL and ORL were < 0.70, so they were excluded as data of interest in the results.

The results of the comparison of mean indices for the Spectralis SD-OCT PP 8 × 8 grid indicate no statistically significant differences in any of the layers between horizontalized and inclined grids (Table 2). Consequently, the inclined grid, obtained automatically in Spectralis SD-OCT, was selected to continue the research. The layer that offers the highest diagnostic yield is RNFL.

### 3.4. Analysis of Cutoff Points for the Mean Indices

After determining the AUROC for each layer, the thickness value (cutoff point) of the mean index was chosen to classify a patient, such that if their thickness was below the selected cutoff point, the patient would be classified as diseased. The criterion for selecting this point was based on choosing the value at which sensitivity and specificity are as equal and high as possible. After selecting the value for each layer, diagnostic validity indices were calculated. Table 3 shows these results.

## 4. Discussion

In this research, the diagnostic ability of the Spectralis SD-OCT PP software with the 8 × 8 grid automatically tilted along the disc-fovea axis at 7° and the grid horizontalized was studied in the thickness of RNFL, GCL, IRL, ORL, complete retina, the ganglion cell complex (RNFL + GCL + IPL), the complex formed by OPLONL, and the complex formed by GCLIPL in the macular area of healthy and glaucomatous subjects. The topographical evaluation of the effect of different states on the thickness of various intraretinal layers was conducted to identify the diagnostic capacity of different changes. Clinical consideration was given to whether it is more effective to use the horizontalized or inclined grid based on the disc-fovea axis. Additionally, a global index was determined for each studied layer, both with the inclined and horizontalized grids, identifying the layer with the highest diagnostic capability. Diagnostic capacities of different topographic patterns were calculated using ROC curves (receiver operating characteristic curve) results. Finally, the diagnostic capability of each studied layer and layer combination was determined after selecting a cutoff point based on sensitivity and specificity to correctly detect the presence of glaucoma. 

In an extensive literature search, no other study following a similar methodology to this research was found, except for the study conducted by Del-Rio-Vellosillo et al. [22], performed by the present research group, where we compared the diagnostic capability of the ganglion cell complex thickness with the 8 × 8 grid of the 7-degree inclined and horizontalized PP algorithm to distinguish between healthy and glaucomatous eyes. 

Numerous authors have studied the influence of the disc-fovea axis on retinal thickness in glaucoma diagnosis. Some authors [23], similarly to this study, found no statistically significant differences in retinal thickness when modifying the disc-fovea axis. In contrast, many other authors did find small differences between both axes, although, like in this research, compensating for the inclination of the disc-fovea axis did not seem to offer clear clinical advantages [24,25,26,27,28,29,30,31,32].

Nouri-Mahdavi et al. [20] compared macular GCLIPL with peripapillary RNFL and confirmed similar results. In the present study, when comparing macular GCLIPL, similar results were obtained with mRNFL and the macular ganglion cell complex. Budenz et al. and Martínez de la Casa et al., similarly to this study, indicated that RNFL has a higher discrimination capability for subjects with glaucoma than other layers [2,33]. However, Rao et al. [34] claimed that IRL parameters are as valid as RNFL. It is noteworthy that the subjects in their study were of Hindu race.

Like some authors [35,36,37,38,39], the present research group found that the thickness of the inner layers of the macula has a higher diagnostic capability than total retinal thickness. Other researchers [29,40,41,42,43,44] have indicated, as it has been observed in the present piece of investigation, that macular thickness and RNFL have high diagnostic sensitivity and specificity to discriminate between healthy and glaucomatous eyes.

Khanal et al. [43] suggested that overall macular thickness could lead to lower sensitivity than segmenting thickness in different layers or layer combinations, in accordance with the results of the present study, as the macula contains regions that are not sensitive to glaucomatous changes. Pazos et al. [42] achieved a high diagnostic capability in the macular ganglion cell complex, although it did not surpass macular RNFL. 

**Figure 5:**
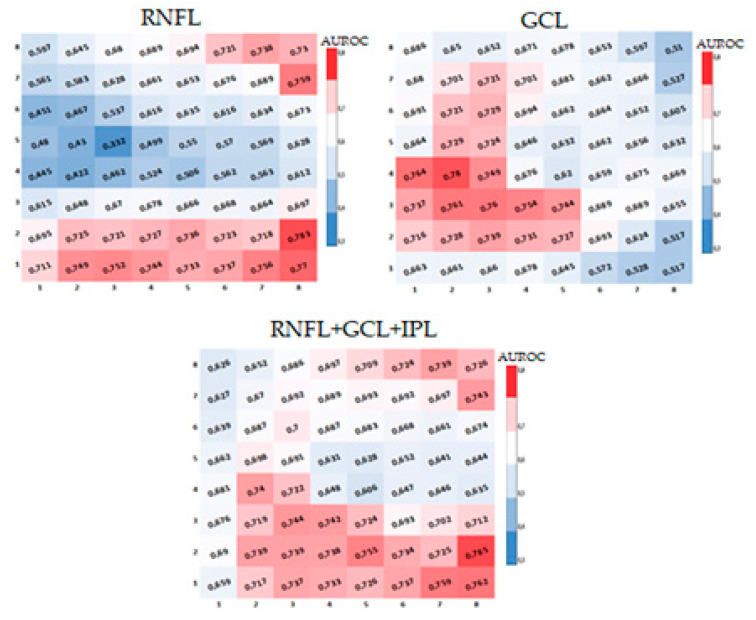
Heatmaps of AUROCs for RNFL, GCL, and the macular ganglion cell complex obtained in this study with the 8 × 8 grid of the Spectralis SD-OCT posterior pole, tilted at 7°, similar to the model described by Hood et al. [14]. Retinal nerve fiber layer (RNFL); ganglion cell layer (GCL); inner plexiform layer (IPL).

Among the different types of existing macular glaucoma diagnostic methods in the literature, there are two that are especially remarkable: the schematic model of glaucomatous damage by Hood et al. [14] and the reduction of intraretinal thickness. Hood et al. created a schematic model of visual field defects in the macula by overlaying OCT-obtained maps onto visual fields. The schematic model predicts the arched defects of initial macular damage and the relatively preserved “central island” in the macula in patients with advanced glaucoma. Numerous authors indicate that to improve sensitivity and specificity, topographic information from visual fields should be combined with OCT images [45,46], analyzing the thickness of pRNFL, macular GCC, and GCL from both optic disc and macular scanning cubes [47,48]. In Figure 5, it can be observed how cells with higher diagnostic capability (cells in red) obtained in this study coincide with the prediction of macular glaucomatous damage defects found by Hood et al. [14]. 

The progression of POAG involves a decrease in intraretinal thickness, and numerous researchers have studied it using various methods. Many authors [49,50,51,52,53] have focused on individual studies of macular RNFL and GCL thickness, while others [12,54], in addition to these studies, have included the photoreceptor layer and the retinal pigment epithelium in their studies. Some authors, however, have focused their work on GCL [4,12,35,36,42,55,56,57,58]. 

Regarding the method of retinal study, Bambo et al. [58] used the ETDRS grid for the analysis of retinal thickness. They indicated that the inner layers of the macula, especially the temporal sector of GCL, provided a good diagnostic capacity for glaucoma. Although the data obtained in this research have coincided, the AUROC analysis results of these authors show greater AUROC values than those found in the present work. However, in another study focusing on the study of RNFL, GCL, and IPL, Garcia-Medina et al. [6] concluded that the PP 8 × 8 asymmetry protocol is superior to the ETDRS protocol in assessing the diagnostic capacity to differentiate between ocular hypertension and POAG, which is why we decided to investigate with the 8 × 8 grid. 

The thickness of the macula in all its regions, as it has been observed in this study, is asymmetric. Ooto et al. [59] also concluded, similarly to the present study, that GCL thickness was significantly lower in the temporal region compared to the nasal region of the macula in glaucoma. Moreover, they created thickness maps that showed vertical asymmetry for all layers, including the inner layers of the retina. 

The results of this research coincide with numerous studies indicating that segmented macular IRL thickness has a greater diagnostic yield than total macular thickness and is similar (though not better) than pRNFL thickness in glaucoma diagnosis [55,60,61]. Most OCT studies that independently or through combinations evaluate the inner layers of the retina in the macula have demonstrated diagnostic accuracy similar to pRNFL thickness. Nevertheless, to date, it is still unknown how the isolated analysis of GCL improves glaucomatous diagnostic capacity [35,36,42,50,55,56,57,58]. In this study, after performing an isolated GCL analysis, it was verified that its diagnostic capacity is not superior to that of other retinal layers, using the 8 × 8 macular grid in Spectralis SD-OCT as the evaluation method.

One may argue that the difference in mean age between healthy and glaucoma patients in this study (Table 1) could affect its results, but it has been shown that a 10-year age difference does not significantly influence either the sensitivity of automated perimetry [62] or retinal thickness measured by OCT [19]. It also was found that there is no difference in IOP between the groups. This result may be attributed to the fact that all participants in the POAG group were under hypotensive topical treatment.

This study has several limitations to consider: predictive values and overall diagnostic accuracy cannot be easily generalized beyond the study population in which they were estimated [63]. Plus, the groups of subjects, both healthy and glaucomatous, consisted of Caucasian patients with POAG, so the results cannot be extrapolated to other ethnicities. In addition, this research is a cross-sectional study, so, due to its nature, it does not allow the study of progressive changes in POAG. Finally, in this study, the degree of glaucoma is not differentiated, which may affect the overall values obtained. To reduce bias and increase diagnostic accuracy, future research should focus on segregating different severity levels. This way, the affected zones and layers could be determined based on the progression and classification of the disease.

## 5. Conclusions

The original findings obtained in this study include the identification of different patterns of macular damage based on the studied layers using a new methodology, a global index indicating the diagnostic capacity of each layer studied in two grids (tilted at 7° and horizontal), and evidence that modifying the orientation of the macular grid does not seem profitable. The retinal layers with the highest diagnostic yield were identified using an objective methodology, along with a cutoff point for retinal thickness in each studied layer facilitating the discrimination of patients with POAG.

Both the heatmap patterns and the results of global index comparisons indicate that the diagnostic capacity of the studied segmentations is similar when considering the 8 × 8 macular grid tilted at 7° or horizontalized. The macular segmentations that offer the highest diagnostic yield in glaucoma discrimination are, in the following order, RNFL, the ganglion cell complex, GCL, GCLIPL, IRL, and the complete retina. In contrast, the thickness of the ORL and OPLONL does not seem useful for discriminating between healthy individuals and those with glaucoma.

To sum up, the 8 × 8 macular grid from the PP software in Spectralis SD-OCT provides a good diagnostic capacity in different layers and combinations of inner retina layers as a complementary method that may be useful for diagnosing POAG.

## Figures and Tables

**Table 1:** Demographic and ophthalmic clinical analysis of the sample. Best corrected visual acuity (BCVA), Intraocular pressure (IOP), standard deviation (SD).

	Group		*Test*	*p*-Value
	Control	Glaucoma		
**Eyes according to sex** *n* (%)			χ^2^ = 0.389	0.533 (chi-squared test)
Men	61 (44.85)	79 (48.5)		
Women	75 (55.15)	84 (51.5)		
**Patients according to sex** *n* (%)			χ^2^ = 1.097	0.295 (chi-squared test)
Men	35 (43.2)	53 (51)		
Women	46 (56.8)	51 (49)		
**Patients according to age** Mean (SD)	66.7 (16.4)	75.5 (12.5)	*t* (181) = −4.13	<0.001 (*t* test)
**Eyes according to age** Mean (SD)	65.4 (16.4)	75.8 (12.2)	*t* (297) = −6.27	<0.001 (*t* test)
**Decimal BCVA.** Mean (SD)	0.9 (0.2)	0.87 (0.55)	*t* (291) = 0.70	0.485 (*t* test)
**IOP** Mean (SD)	17.22 (3.04)	17.13 (3.75)	*t* (297) = 0.23	0.819 (*t* test)
**Vertical optic disc cupping** Mean (SD)	0.39 (0.25)	0.57 (0.25)	*t* (259) = −5.77	<0.001 (*t* test)
**Mean deviation.** Mean (SD)	−1.13 (1.38)	−6.99 (7.11)	*t* (257) = 7.97	<0.001 (*t* test)
**Pattern standard deviation** Mean (SD)	1.84 (0.71)	5.47 (3.69)	*t* (257) = −9.51	<0.001 (*t* test)

**Table 2:** Comparison of the mean index between the inclined and horizontal grids of the 8 × 8 grid of the posterior pole in the layers and layer combinations of the studied macular retina: complete retina, retinal nerve fiber layer (RNFL), ganglion cell layer (GCL), ganglion cell complex, combination formed by the ganglion cell layer and the inner plexiform layer (GCLIPL), and inner retinal layers (IRL).

	Position of the Grid. AUROC (CI95%)		Comparison	
Segmentation	Horizontalized	Inclined	*Z*	*p*-Value (DeLong Test)
**Complete retina**	0.743 (0.688–0.798)	0.752 (0.698–0.806)	1.594	0.111
**RNFL**	0.794 (0.744–0.843)	0.796 (0.746–0.845)	0.821	0.412
**GCL**	0.781 (0.729–0.834)	0.784 (0.732–0.836)	0.558	0.577
**Ganglion cell complex**	0.784 (0.733–0.835)	0.785 (0.734–0.835)	0.195	0.845
**GCLIPL**	0.769 (0.715–0.823)	0.770 (0.717–0.824)	0.405	0.685
**IRL**	0.751 (0.697–0.806)	0.755 (0.701–0.809)	0.886	0.376

**Table 3:** Cutoff points of the mean index (in microns) for classifying a patient as healthy (if the thickness is above the cutoff point) or as glaucomatous (if the thickness is below the cutoff point), and diagnostic capability indices for different retinal layers and layer combinations. Retinal nerve fiber layer (RNFL), ganglion cell layer (GCL), ganglion cell complex, combination formed by the ganglion cell layer and the inner plexiform layer (GCLIPL), and inner retinal layers (IRL).

Segmentation	Cuttof Point (Microns)	Specificity % (CI95%)	Sensitivity % (CI95%)	PPV % (CI95%)	NPV % (CI95%)
**Complete retina**	286.00	64.42 (56.76–72.07)	74.26 (66.55–81.98)	75 (67.47–82.53)	63.52 (55.73–71.32)
**RNFL**	52.24	71.17 (63.9–78.43)	72.79 (64.95–80.64)	75.82 (68.71–82.93)	67.81 (59.89–75.73)
**GCL**	32.40	71.17 (63.9–78.43)	72.79 (64.95–80.64)	75.82 (68.71–82.93)	67.81 (59.89–75.73)
**Ganglion cell complex**	96.21	66.26 (58.69–73.82)	74.26 (66.55–81.98)	75.52 (68.13–82.92)	64.74 (56.93–72.56)
**GCLIPL**	62.85	72.39 (65.22–79.56)	72.79 (64.95–80.64)	76.13 (69.1–83.16)	68.75 (60.83–76.67)
**IRL**	210.50	65.03 (57.4–72.66)	72.06 (64.15–79.97)	73.61 (66.07–81.16)	63.23 (55.31–71.14)

## Data Availability

The data presented in this study are available on reasonable request from the corresponding author.

## Supplementary Materials

The following supporting information can be downloaded at: https://www.mdpi.com/article/10.3390/jcm13041016/s1. Table S1. Area Under the Curve (AUROC) value, 95% Confidence Interval (CI) and p-value for each cell of the 8 × 8 macular grid of the macular Retinal Nerve Fiber Layer (RNFL) with the grid tilted at 7° and with the grid horizontalized. Table S2. Area Under the Receiver Operating Characteristic Curve (AUROC) value, 95% Confidence Interval (CI) and p-value for each cell of the 8 × 8 macular grid of the Ganglion Cell Layer (GCL) with the grid tilted at 7° and with the grid horizontalized. Table S3. Area Under the Receiver Operating Characteristic Curve (AUROC) value, 95% Confidence Interval (CI) and p-value for each cell of the 8 × 8 macular grid of the entire retina with the grid tilted at 7° and with the grid horizontalized. Table S4. Area Under the Receiver Operating Characteristic Curve (AUROC) value, 95% Confidence Interval (CI) and p-value for each cell of the 8 × 8 macular grid of the ganglion cell complex (RNFL+GCL+IPL) with the grid tilted at 7° and with the grid horizontalized. Table S5. Area Under the Receiver Operating Characteristic Curve (AUROC) value, 95% Confidence Interval (CI) and p-value for each cell of the 8 × 8 macular grid of the ganglion cell layer+inner plexiform layer (GCLIPL) with the grid tilted at 7° and with the grid horizontalized. Table S6. Area Under the Receiver Operating Characteristic Curve (AUROC) value, 95% Confidence Interval (CI) and p-value for each cell of the 8 × 8 macular grid of the outer plexiform layer+outer nuclear layer (OPLONL) with the grid tilted at 7° and with the grid horizontalized. Table S7. Area Under the Receiver Operating Characteristic Curve (AUROC) value, 95% Confidence Interval (CI) and p-value for each cell of the 8 × 8 macular grid of the inner retinal layers (INL) with the grid tilted at 7° and with the grid horizontalized. Table S8. Area Under the Receiver Operating Characteristic Curve (AUROC) value, 95% Confidence Interval (CI) and p-value for each cell of the 8 × 8 macular grid of the outer retinal layer (ORL) with the grid tilted at 7° and with the grid horizontalized. Table S9. AUROC and comparison of mean and weighted indices for the 8 × 8 grid of the Posterior Pole (PP) using Spectralis SD-OCT in the thickness of the complete retina. Table S10. AUROC and comparison of mean and weighted indices for the 8 × 8 grid of the Posterior Pole (PP) using Spectralis SD-OCT in the thickness of the RNFL. Table S11. AUROC and comparison of mean and weighted indices for the 8 × 8 grid of the Posterior Pole (PP) using Spectralis SD-OCT in the thickness of the GCL. Table S12. AUROC and comparison of mean and weighted indices for the 8 × 8 grid of the Posterior Pole (PP) using Spectralis SD-OCT in the thickness of the ganglion cell complex. Table S13. AUROC and comparison of mean and weighted indices for the 8 × 8 grid of the Posterior Pole (PP) using Spectralis SD-OCT in the thickness of GCLIPL. Table S14. AUROC and comparison of mean and weighted indices for the 8 × 8 grid of the Posterior Pole (PP) using Spectralis SD-OCT in the thickness of the IRL.
