# Supplementary material for: Evaluation of the Diagnostic Capability of Spectralis SD-OCT 8 × 8 Posterior Pole Software with the Grid Tilted at 7 Degrees and Horizontalized in Glaucoma
Source: J Clin Med. 2024 Feb 9;13(4):1016. doi: 10.3390/jcm13041016 (PMC10889121; doi:10.3390/jcm13041016)
Supplement: Supplementary file 1 [file jcm-13-01016-s001.zip › jcm-2826127-supplementary.pdf]

Supplementary

Table S1. Area Under the Curve (AUROC) value, 95% Confidence Interval (CI) and p-value for each cell of the 8x8 macular grid of the macular Retinal Nerve Fiber Layer (RNFL) with the grid tilted at 7° and with the grid horizontalized.

	INCLINED 7°			HORIZONTAL		
	AUROC	CI 95%	*p*-value	AUROC	CI 95%	*p*-value
RNFL _1.1	0.711	0.652 - 0.769	< 0.001	0.711	0.653 - 0.77	< 0.001
RNFL _1.2	0.749	0.693 - 0.805	< 0.001	0.74	0.683 - 0.796	< 0.001
RNFL _1.3	0.752	0.697 - 0.808	< 0.001	0.763	0.709 - 0.818	< 0.001
RNFL _1.4	0.744	0.689 - 0.8	< 0.001	0.741	0.685 - 0.797	< 0.001
RNFL _1.5	0.733	0.677 - 0.79	< 0.001	0.737	0.68 - 0.793	< 0.001
RNFL _1.6	0.737	0.681 - 0.794	< 0.001	0.73	0.674 - 0.787	< 0.001
RNFL _1.7	0.756	0.702 - 0.811	< 0.001	0.758	0.703 - 0.812	< 0.001
RNFL _1.8	0.77	0.716 - 0.823	< 0.001	0.74	0.683 - 0.796	< 0.001
RNFL _2.1	0.695	0.635 - 0.756	< 0.001	0.664	0.601 - 0.726	< 0.001
RNFL _2.2	0.725	0.667 - 0.783	< 0.001	0.711	0.652 - 0.77	< 0.001
RNFL _2.3	0.721	0.662 - 0.779	< 0.001	0.72	0.661 - 0.779	< 0.001
RNFL _2.4	0.727	0.67 - 0.785	< 0.001	0.717	0.659 - 0.775	< 0.001
RNFL _2.5	0.736	0.68 - 0.793	< 0.001	0.737	0.681 - 0.794	< 0.001
RNFL _2.6	0.723	0.665 - 0.781	< 0.001	0.738	0.682 - 0.795	< 0.001
RNFL _2.7	0.718	0.66 - 0.777	< 0.001	0.735	0.678 - 0.792	< 0.001
RNFL _2.8	0.783	0.731 - 0.835	< 0.001	0.787	0.735 - 0.838	< 0.001
RNFL _3.1	0.615	0.55 - 0.679	0.001	0.543	0.477 - 0.609	0.205
RNFL _3.2	0.648	0.585 - 0.711	< 0.001	0.594	0.528 - 0.659	0.006
RNFL 3.3	0.67	0.608 - 0.731	< 0.001	0.65	0.587 - 0.713	< 0.001
RNFL _3.4	0.678	0.617 - 0.739	< 0.001	0.675	0.614 - 0.736	< 0.001
RNFL _3.5	0.666	0.604 - 0.728	< 0.001	0.669	0.608 - 0.731	< 0.001
RNFL _3.6	0.668	0.606 - 0.729	< 0.001	0.676	0.615 - 0.737	< 0.001
RNFL _3.7	0.664	0.602 - 0.725	< 0.001	0.674	0.613 - 0.735	< 0.001
RNFL _3.8	0.697	0.638 - 0.757	< 0.001	0.71	0.652 - 0.769	< 0.001
RNFL _4.1	0.445	0.379 - 0.512	0.109	0.434	0.368 - 0.5	0.054
RNFL 4.2	0.422	0.356 - 0.487	0.022	0.411	0.346 - 0.476	0.009
RNFL 4.3	0.462	0.396 - 0.528	0.268	0.371	0.307 - 0.434	< 0.001
RNFL _4.4	0.524	0.458 - 0.591	0.473	0.509	0.443 - 0.575	0.79
RNFL 4.5	0.506	0.44 - 0.572	0.86	0.503	0.436 - 0.569	0.936
RNFL _4.6	0.562	0.496 - 0.628	0.07	0.576	0.511 - 0.642	0.025
RNFL _4.7	0.563	0.497 - 0.629	0.065	0.597	0.532 - 0.662	0.004
RNFL _4.8	0.612	0.548 - 0.676	0.001	0.633	0.569 - 0.696	< 0.001
RNFL_5.1	0.48	0.414 - 0.547	0.558	0.479	0.412 - 0.545	0.528
RNFL_5.2	0.43	0.364 - 0.496	0.039	0.424	0.359 - 0.49	0.026

RNFL _5.3	0.332	0.27 - 0.394	< 0.001	0.347	0.284 - 0.41	< 0.001
RNFL _5.4	0.499	0.433 - 0.566	0.98	0.519	0.453 - 0.585	0.577
RNFL _5.5	0.55	0.485 - 0.616	0.138	0.542	0.476 - 0.608	0.218
RNFL _5.6	0.57	0.504 - 0.636	0.04	0.565	0.499 - 0.631	0.056
RNFL _5.7	0.569	0.504 - 0.635	0.042	0.555	0.489 - 0.621	0.107
RNFL _5.8	0.628	0.564 - 0.691	< 0.001	0.607	0.543 - 0.671	0.002
RNFL _6.1	0.451	0.385 - 0.517	0.15	0.479	0.412 - 0.546	0.538
RNFL _6.2	0.467	0.4 - 0.533	0.328	0.505	0.438 - 0.572	0.88
RNFL _6.3	0.537	0.471 - 0.604	0.276	0.564	0.498 - 0.63	0.06
RNFL _6.4	0.616	0.552 - 0.68	0.001	0.622	0.559 - 0.686	< 0.001
RNFL _6.5	0.635	0.572 - 0.699	< 0.001	0.625	0.562 - 0.689	< 0.001
RNFL _6.6	0.616	0.552 - 0.68	0.001	0.597	0.533 - 0.662	0.004
RNFL _6.7	0.634	0.57 - 0.697	< 0.001	0.607	0.542 - 0.671	0.002
RNFL _6.8	0.673	0.611 - 0.734	< 0.001	0.662	0.601 - 0.724	< 0.001
RNFL _7.1	0.561	0.495 - 0.627	0.074	0.603	0.538 - 0.667	0.003
RNFL _7.2	0.583	0.517 - 0.648	0.015	0.62	0.556 - 0.685	< 0.001
RNFL _7.3	0.628	0.564 - 0.691	< 0.001	0.646	0.584 - 0.709	< 0.001
RNFL _7.4	0.661	0.6 - 0.723	< 0.001	0.661	0.599 - 0.723	< 0.001
RNFL _7.5	0.653	0.59 - 0.715	< 0.001	0.654	0.592 - 0.716	< 0.001
RNFL _7.6	0.676	0.615 - 0.737	< 0.001	0.668	0.607 - 0.73	< 0.001
RNFL _7.7	0.689	0.629 - 0.749	< 0.001	0.687	0.627 - 0.747	< 0.001
RNFL _7.8	0.759	0.705 - 0.813	< 0.001	0.733	0.676 - 0.789	< 0.001
RNFL _8.1	0.597	0.532 - 0.662	0.004	0.624	0.56 - 0.688	< 0.001
RNFL _8.2	0.645	0.583 - 0.708	< 0.001	0.667	0.605 - 0.729	< 0.001
RNFL _8.3	0.68	0.619 - 0.74	< 0.001	0.684	0.624 - 0.745	< 0.001
RNFL _8.4	0.689	0.629 - 0.749	< 0.001	0.694	0.634 - 0.753	< 0.001
RNFL _8.5	0.694	0.635 - 0.753	< 0.001	0.705	0.647 - 0.764	< 0.001
RNFL_ 8.6	0.721	0.664 - 0.778	< 0.001	0.717	0.659 - 0.775	< 0.001
RNFL _8.7	0.738	0.682 - 0.794	< 0.001	0.744	0.688 - 0.799	< 0.001
RNFL _8.8	0.73	0.673 - 0.787	< 0.001	0.759	0.705 - 0.814	< 0.001

Table S2. Area Under the Receiver Operating Characteristic Curve (AUROC) value, 95% Confidence Interval (CI) and p-value for each cell of the 8x8 macular grid of the Ganglion Cell Layer (GCL) with the grid tilted at 7° and with the grid horizontalized.

	INCLINED 7°			HORIZONTAL		
	AUROC	CI 95%	p-value	AUROC	CI 95%	p-value
GCL_1.1	0.663	0.6 - 0.725	< 0.001	0.662	0.6 - 0.724	< 0.001
GCL _1.2	0.661	0.599 - 0.724	< 0.001	0.684	0.623 - 0.744	< 0.001
GCL _1.3	0.66	0.598 - 0.722	< 0.001	0.681	0.62 - 0.742	< 0.001
GCL _1.4	0.678	0.616 - 0.739	< 0.001	0.681	0.62 - 0.742	< 0.001
GCL _1.5	0.645	0.582 - 0.708	< 0.001	0.664	0.602 - 0.727	< 0.001
GCL _1.6	0.572	0.506 - 0.638	0.035	0.596	0.53 - 0.661	0.005
GCL _1.7	0.528	0.461 - 0.595	0.412	0.506	0.439 - 0.572	0.863
GCL _1.8	0.517	0.451 - 0.583	0.617	0.52	0.453 - 0.586	0.56
GCL _2.1	0.716	0.658 - 0.775	< 0.001	0.717	0.659 - 0.776	< 0.001
GCL _2.2	0.728	0.671 - 0.786	< 0.001	0.728	0.671 - 0.786	< 0.001
GCL _2.3	0.739	0.682 - 0.795	< 0.001	0.746	0.69 - 0.802	< 0.001
GCL _2.4	0.731	0.674 - 0.789	< 0.001	0.729	0.671 - 0.787	< 0.001
GCL _2.5	0.727	0.668 - 0.786	< 0.001	0.725	0.666 - 0.784	< 0.001
GCL _2.6	0.693	0.632 - 0.753	< 0.001	0.693	0.633 - 0.754	< 0.001
GCL _2.7	0.624	0.56 - 0.688	< 0.001	0.627	0.562 - 0.691	< 0.001
GCL _2.8	0.517	0.45 - 0.584	0.616	0.481	0.414 - 0.548	0.574
GCL _3.1	0.737	0.68 - 0.793	< 0.001	0.746	0.689 - 0.802	< 0.001
GCL _3.2	0.761	0.706 - 0.815	< 0.001	0.767	0.712 - 0.821	< 0.001
GCL _3.3	0.76	0.706 - 0.815	< 0.001	0.764	0.709 - 0.819	< 0.001
GCL _3.4	0.754	0.698 - 0.809	< 0.001	0.748	0.693 - 0.804	< 0.001
GCL _3.5	0.744	0.688 - 0.801	< 0.001	0.752	0.696 - 0.808	< 0.001
GCL _3.6	0.689	0.628 - 0.75	< 0.001	0.692	0.631 - 0.753	< 0.001
GCL _3.7	0.689	0.629 - 0.75	< 0.001	0.68	0.619 - 0.741	< 0.001
GCL _3.8	0.655	0.592 - 0.718	< 0.001	0.64	0.576 - 0.703	< 0.001
GCL _4.1	0.764	0.709 - 0.818	< 0.001	0.735	0.679 - 0.792	< 0.001
GCL _4.2	0.78	0.727 - 0.833	< 0.001	0.764	0.71 - 0.819	< 0.001
GCL _4.3	0.749	0.693 - 0.805	< 0.001	0.747	0.691 - 0.803	< 0.001
GCL _4.4	0.676	0.615 - 0.737	< 0.001	0.679	0.619 - 0.74	< 0.001
GCL _4.5	0.62	0.557 - 0.684	< 0.001	0.627	0.563 - 0.69	< 0.001
GCL _4.6	0.659	0.596 - 0.721	< 0.001	0.66	0.597 - 0.722	< 0.001
GCL _4.7	0.675	0.614 - 0.736	< 0.001	0.678	0.617 - 0.739	< 0.001
GCL _4.8	0.669	0.607 - 0.73	< 0.001	0.691	0.63 - 0.751	< 0.001
GCL _5.1	0.664	0.603 - 0.726	< 0.001	0.657	0.595 - 0.719	< 0.001
GCL _5.2	0.729	0.672 - 0.787	< 0.001	0.711	0.653 - 0.77	< 0.001

GCL _5.3	0.724	0.666 - 0.781	< 0.001	0.714	0.656 - 0.773	< 0.001
GCL _5.4	0.646	0.583 - 0.71	< 0.001	0.643	0.58 - 0.706	< 0.001
GCL _5.5	0.632	0.569 - 0.696	< 0.001	0.621	0.557 - 0.685	< 0.001
GCL _5.6	0.662	0.6 - 0.724	< 0.001	0.655	0.592 - 0.718	< 0.001
GCL _5.7	0.656	0.593 - 0.718	< 0.001	0.653	0.591 - 0.716	< 0.001
GCL _5.8	0.632	0.569 - 0.696	< 0.001	0.647	0.584 - 0.709	< 0.001
GCL _6.1	0.691	0.631 - 0.751	< 0.001	0.703	0.643 - 0.762	< 0.001
GCL _6.2	0.721	0.663 - 0.779	< 0.001	0.724	0.667 - 0.782	< 0.001
GCL _6.3	0.729	0.672 - 0.787	< 0.001	0.723	0.665 - 0.781	< 0.001
GCL _6.4	0.694	0.634 - 0.754	< 0.001	0.686	0.626 - 0.747	< 0.001
GCL _6.5	0.662	0.6 - 0.724	< 0.001	0.664	0.602 - 0.726	< 0.001
GCL _6.6	0.664	0.602 - 0.726	< 0.001	0.664	0.602 - 0.726	< 0.001
GCL _6.7	0.652	0.59 - 0.715	< 0.001	0.648	0.586 - 0.711	< 0.001
GCL _6.8	0.605	0.54 - 0.669	0.002	0.61	0.546 - 0.674	0.001
GCL _7.1	0.68	0.62 - 0.741	< 0.001	0.695	0.635 - 0.755	< 0.001
GCL _7.2	0.701	0.641 - 0.76	< 0.001	0.695	0.635 - 0.755	< 0.001
GCL _7.3	0.721	0.664 - 0.779	< 0.001	0.716	0.657 - 0.774	< 0.001
GCL _7.4	0.701	0.641 - 0.76	< 0.001	0.695	0.636 - 0.755	< 0.001
GCL _7.5	0.681	0.62 - 0.742	< 0.001	0.672	0.61 - 0.734	< 0.001
GCL _7.6	0.662	0.6 - 0.724	< 0.001	0.66	0.598 - 0.722	< 0.001
GCL _7.7	0.666	0.604 - 0.727	< 0.001	0.623	0.559 - 0.687	< 0.001
GCL _7.8	0.527	0.46 - 0.593	0.432	0.537	0.471 - 0.603	0.274
GCL _8.1	0.686	0.625 - 0.747	< 0.001	0.643	0.58 - 0.707	< 0.001
GCL _8.2	0.65	0.587 - 0.713	< 0.001	0.651	0.589 - 0.714	< 0.001
GCL _8.3	0.652	0.589 - 0.714	< 0.001	0.665	0.603 - 0.726	< 0.001
GCL _8.4	0.671	0.609 - 0.732	< 0.001	0.666	0.604 - 0.727	< 0.001
GCL _8.5	0.678	0.617 - 0.739	< 0.001	0.681	0.62 - 0.742	< 0.001
GCL _8.6	0.653	0.591 - 0.715	< 0.001	0.658	0.596 - 0.72	< 0.001
GCL _8.7	0.597	0.531 - 0.662	0.005	0.615	0.551 - 0.679	0.001
GCL _8.8	0.51	0.443 - 0.577	0.773	0.47	0.404 - 0.537	0.382

Table S3. Area Under the Receiver Operating Characteristic Curve (AUROC) value, 95% Confidence Interval (CI) and p-value for each cell of the 8x8 macular grid of the entire retina with the grid tilted at 7° and with the grid horizontalized.

	INCLINED 7°			HORIZONTAL		
	**AUROC**	**CI 95%**	***p*-value**	**AUROC**	**CI 95%**	***p*-value**
RETINA_1.1	0.594	0.529 - 0.659	0.006	0.6	0.535 - 0.665	0.003
RETINA_1.2	0.622	0.558 - 0.686	< 0.001	0.609	0.544 - 0.673	0.001
RETINA_1.3	0.66	0.598 - 0.722	< 0.001	0.662	0.6 - 0.724	< 0.001
RETINA_1.4	0.672	0.61 - 0.733	< 0.001	0.667	0.605 - 0.729	< 0.001
RETINA_1.5	0.682	0.621 - 0.743	< 0.001	0.676	0.614 - 0.737	< 0.001
RETINA_1.6	0.706	0.648 - 0.765	< 0.001	0.693	0.634 - 0.753	< 0.001
RETINA_1.7	0.726	0.668 - 0.783	< 0.001	0.722	0.664 - 0.779	< 0.001
RETINA_1.8	0.719	0.661 - 0.777	< 0.001	0.711	0.652 - 0.769	< 0.001
RETINA_2.1	0.606	0.541 - 0.67	0.002	0.602	0.537 - 0.666	0.003
RETINA_2.2	0.648	0.585 - 0.711	< 0.001	0.635	0.571 - 0.698	< 0.001
RETINA_2.3	0.665	0.603 - 0.726	< 0.001	0.661	0.599 - 0.723	< 0.001
RETINA_2.4	0.657	0.595 - 0.719	< 0.001	0.652	0.589 - 0.714	< 0.001
RETINA_2.5	0.671	0.609 - 0.733	< 0.001	0.666	0.604 - 0.728	< 0.001
RETINA_2.6	0.683	0.622 - 0.744	< 0.001	0.686	0.625 - 0.746	< 0.001
RETINA_2.7	0.701	0.642 - 0.761	< 0.001	0.705	0.646 - 0.764	< 0.001
RETINA_2.8	0.763	0.709 - 0.817	< 0.001	0.752	0.697 - 0.808	< 0.001
RETINA_3.1	0.603	0.538 - 0.668	0.003	0.584	0.519 - 0.649	0.014
RETINA_3.2	0.64	0.577 - 0.703	< 0.001	0.627	0.563 - 0.69	< 0.001
RETINA_3.3	0.652	0.59 - 0.715	< 0.001	0.648	0.585 - 0.711	< 0.001
RETINA_3.4	0.669	0.607 - 0.731	< 0.001	0.661	0.599 - 0.724	< 0.001
RETINA_3.5	0.654	0.591 - 0.716	< 0.001	0.666	0.604 - 0.728	< 0.001
RETINA_3.6	0.634	0.571 - 0.698	< 0.001	0.641	0.578 - 0.704	< 0.001
RETINA_3.7	0.661	0.599 - 0.723	< 0.001	0.664	0.603 - 0.726	< 0.001
RETINA_3.8	0.689	0.629 - 0.748	< 0.001	0.711	0.652 - 0.769	< 0.001
RETINA_4.1	0.58	0.515 - 0.645	0.019	0.567	0.501 - 0.632	0.05
RETINA_4.2	0.623	0.559 - 0.686	< 0.001	0.604	0.539 - 0.668	0.002
RETINA_4.3	0.629	0.565 - 0.692	< 0.001	0.616	0.552 - 0.68	0.001
RETINA_4.4	0.567	0.501 - 0.633	0.05	0.561	0.496 - 0.627	0.071
RETINA_4.5	0.534	0.467 - 0.6	0.325	0.54	0.474 - 0.606	0.243
RETINA_4.6	0.573	0.507 - 0.638	0.032	0.576	0.51 - 0.641	0.026
RETINA_4.7	0.595	0.53 - 0.66	0.005	0.611	0.546 - 0.675	0.001
RETINA_4.8	0.608	0.544 - 0.673	0.001	0.638	0.574 - 0.701	< 0.001
RETINA_5.1	0.555	0.489 - 0.621	0.107	0.557	0.491 - 0.623	0.094
RETINA_5.2	0.577	0.511 - 0.642	0.025	0.582	0.517 - 0.648	0.015
RETINA_5.3	0.591	0.526 - 0.656	0.008	0.595	0.53 - 0.66	0.005
RETINA_5.4	0.542	0.476 - 0.608	0.217	0.542	0.476 - 0.608	0.216

RETINA_5.5	0.548	0.482 - 0.614	0.162	0.544	0.478 - 0.61	0.199
RETINA_5.6	0.566	0.5 - 0.631	0.054	0.563	0.497 - 0.629	0.065
RETINA_5.7	0.578	0.513 - 0.643	0.022	0.575	0.509 - 0.64	0.028
RETINA_5.8	0.602	0.538 - 0.667	0.003	0.587	0.522 - 0.653	0.01
RETINA_6.1	0.563	0.497 - 0.629	0.066	0.575	0.509 - 0.641	0.028
RETINA_6.2	0.601	0.536 - 0.665	0.003	0.606	0.541 - 0.67	0.002
RETINA_6.3	0.615	0.552 - 0.679	0.001	0.616	0.552 - 0.68	0.001
RETINA_6.4	0.611	0.547 - 0.676	0.001	0.612	0.548 - 0.676	0.001
RETINA_6.5	0.6	0.536 - 0.665	0.003	0.596	0.532 - 0.661	0.005
RETINA_6.6	0.607	0.542 - 0.671	0.002	0.599	0.534 - 0.663	0.004
RETINA_6.7	0.624	0.56 - 0.687	< 0.001	0.604	0.54 - 0.669	0.002
RETINA_6.8	0.641	0.578 - 0.704	< 0.001	0.637	0.574 - 0.7	< 0.001
RETINA_7.1	0.57	0.505 - 0.636	0.039	0.58	0.514 - 0.645	0.019
RETINA_7.2	0.61	0.545 - 0.674	0.001	0.608	0.544 - 0.673	0.001
RETINA_7.3	0.624	0.56 - 0.688	< 0.001	0.62	0.556 - 0.684	< 0.001
RETINA_7.4	0.624	0.56 - 0.688	< 0.001	0.629	0.565 - 0.692	< 0.001
RETINA_7.5	0.633	0.569 - 0.696	< 0.001	0.63	0.566 - 0.693	< 0.001
RETINA_7.6	0.635	0.572 - 0.698	< 0.001	0.634	0.571 - 0.697	< 0.001
RETINA_7.7	0.665	0.604 - 0.727	< 0.001	0.662	0.6 - 0.724	< 0.001
RETINA_7.8	0.704	0.646 - 0.763	< 0.001	0.684	0.624 - 0.745	< 0.001
RETINA_8.1	0.58	0.514 - 0.645	0.019	0.588	0.523 - 0.653	0.01
RETINA_8.2	0.603	0.538 - 0.667	0.003	0.62	0.556 - 0.684	< 0.001
RETINA_8.3	0.639	0.576 - 0.702	< 0.001	0.645	0.583 - 0.708	< 0.001
RETINA_8.4	0.648	0.585 - 0.71	< 0.001	0.655	0.593 - 0.718	< 0.001
RETINA_8.5	0.666	0.604 - 0.727	< 0.001	0.673	0.612 - 0.734	< 0.001
RETINA_8.6	0.689	0.629 - 0.749	< 0.001	0.69	0.63 - 0.749	< 0.001
RETINA_8.7	0.706	0.648 - 0.765	< 0.001	0.706	0.647 - 0.764	< 0.001
RETINA_8.8	0.698	0.639 - 0.757	< 0.001	0.707	0.648 - 0.766	< 0.001

Table S4. Area Under the Receiver Operating Characteristic Curve (AUROC) value, 95% Confidence Interval (CI) and p-value for each cell of the 8x8 macular grid of the ganglion cell complex (RNFL+GCL+IPL) with the grid tilted at 7° and with the grid horizontalized.

	INCLINED 7°			HORIZONTAL		
	**AUROC**	**CI 95%**	***p*-value**	**AUROC**	**CI 95%**	***p*-value**
RNFL+GCL+IPL_1.1	0.659	0.597 - 0.722	< 0.001	0.66	0.597 - 0.722	< 0.001
RNFL+GCL+IPL _1.2	0.717	0.659 - 0.775	< 0.001	0.712	0.654 - 0.771	< 0.001
RNFL+GCL+IPL _1.3	0.737	0.681 - 0.794	< 0.001	0.744	0.688 - 0.801	< 0.001
RNFL+GCL+IPL _1.4	0.733	0.676 - 0.79	< 0.001	0.728	0.671 - 0.786	< 0.001
RNFL+GCL+IPL _1.5	0.726	0.668 - 0.783	< 0.001	0.724	0.667 - 0.782	< 0.001
RNFL+GCL+IPL _1.6	0.737	0.68 - 0.793	< 0.001	0.722	0.664 - 0.779	< 0.001
RNFL+GCL+IPL _1.7	0.759	0.704 - 0.813	< 0.001	0.76	0.706 - 0.814	< 0.001
RNFL+GCL+IPL _1.8	0.762	0.707 - 0.816	< 0.001	0.743	0.687 - 0.799	< 0.001
RNFL+GCL+IPL _2.1	0.69	0.63 - 0.751	< 0.001	0.682	0.62 - 0.743	< 0.001
RNFL+GCL+IPL _2.2	0.739	0.682 - 0.796	< 0.001	0.716	0.657 - 0.774	< 0.001
RNFL+GCL+IPL _2.3	0.739	0.683 - 0.796	< 0.001	0.745	0.689 - 0.801	< 0.001
RNFL+GCL+IPL _2.4	0.738	0.682 - 0.795	< 0.001	0.729	0.671 - 0.786	< 0.001
RNFL+GCL+IPL _2.5	0.755	0.699 - 0.81	< 0.001	0.751	0.696 - 0.807	< 0.001
RNFL+GCL+IPL _2.6	0.734	0.677 - 0.791	< 0.001	0.739	0.683 - 0.796	< 0.001
RNFL+GCL+IPL _2.7	0.725	0.667 - 0.783	< 0.001	0.737	0.68 - 0.793	< 0.001
RNFL+GCL+IPL _2.8	0.785	0.733 - 0.837	< 0.001	0.778	0.726 - 0.831	< 0.001
RNFL+GCL+IPL _3.1	0.676	0.615 - 0.737	< 0.001	0.674	0.613 - 0.735	< 0.001
RNFL+GCL+IPL _3.2	0.719	0.661 - 0.777	< 0.001	0.72	0.661 - 0.778	< 0.001
RNFL+GCL+IPL _3.3	0.744	0.688 - 0.8	< 0.001	0.742	0.685 - 0.799	< 0.001
RNFL+GCL+IPL _3.4	0.742	0.685 - 0.798	< 0.001	0.737	0.681 - 0.794	< 0.001
RNFL+GCL+IPL _3.5	0.724	0.665 - 0.782	< 0.001	0.726	0.669 - 0.784	< 0.001
RNFL+GCL+IPL _3.6	0.693	0.633 - 0.754	< 0.001	0.698	0.638 - 0.758	< 0.001
RNFL+GCL+IPL _3.7	0.702	0.642 - 0.761	< 0.001	0.706	0.647 - 0.765	< 0.001
RNFL+GCL+IPL _3.8	0.712	0.653 - 0.77	< 0.001	0.727	0.67 - 0.784	< 0.001
RNFL+GCL+IPL _4.1	0.681	0.62 - 0.742	< 0.001	0.677	0.616 - 0.738	< 0.001
RNFL+GCL+IPL _4.2	0.74	0.684 - 0.797	< 0.001	0.728	0.67 - 0.786	< 0.001
RNFL+GCL+IPL _4.3	0.722	0.664 - 0.779	< 0.001	0.71	0.651 - 0.769	< 0.001
RNFL+GCL+IPL _4.4	0.648	0.585 - 0.711	< 0.001	0.647	0.584 - 0.71	< 0.001
RNFL+GCL+IPL_4.5	0.606	0.541 - 0.67	0.002	0.609	0.545 - 0.674	0.001
RNFL+GCL+IPL_4.6	0.647	0.584 - 0.71	< 0.001	0.648	0.585 - 0.711	< 0.001
RNFL+GCL+IPL _4.7	0.646	0.582 - 0.709	< 0.001	0.657	0.594 - 0.719	< 0.001
RNFL+GCL+IPL _4.8	0.635	0.572 - 0.698	< 0.001	0.656	0.594 - 0.718	< 0.001
RNFL+GCL+IPL _5.1	0.662	0.601 - 0.724	< 0.001	0.655	0.593 - 0.717	< 0.001
RNFL+GCL+IPL _5.2	0.698	0.638 - 0.758	< 0.001	0.688	0.628 - 0.748	< 0.001
RNFL+GCL+IPL _5.3	0.691	0.631 - 0.752	< 0.001	0.688	0.627 - 0.748	< 0.001
RNFL+GCL+IPL _5.4	0.631	0.568 - 0.695	< 0.001	0.631	0.567 - 0.694	< 0.001

RNFL+GCL+IPL _5.5	0.628	0.564 - 0.692	< 0.001	0.616	0.552 - 0.68	0.001
RNFL+GCL+IPL_5.6	0.652	0.589 - 0.714	< 0.001	0.647	0.584 - 0.71	< 0.001
RNFL+GCL+IPL_5.7	0.641	0.578 - 0.704	< 0.001	0.639	0.576 - 0.703	< 0.001
RNFL+GCL+IPL _5.8	0.644	0.581 - 0.707	< 0.001	0.636	0.573 - 0.699	< 0.001
RNFL+GCL+IPL _6.1	0.639	0.577 - 0.702	< 0.001	0.641	0.579 - 0.704	< 0.001
RNFL+GCL+IPL _6.2	0.687	0.627 - 0.747	< 0.001	0.685	0.625 - 0.745	< 0.001
RNFL+GCL+IPL _6.3	0.7	0.641 - 0.76	< 0.001	0.691	0.631 - 0.752	< 0.001
RNFL+GCL+IPL _6.4	0.687	0.627 - 0.748	< 0.001	0.687	0.626 - 0.748	< 0.001
RNFL+GCL+IPL _6.5	0.683	0.622 - 0.744	< 0.001	0.678	0.616 - 0.739	< 0.001
RNFL+GCL+IPL _6.6	0.668	0.607 - 0.73	< 0.001	0.657	0.595 - 0.719	< 0.001
RNFL+GCL+IPL _6.7	0.661	0.599 - 0.723	< 0.001	0.642	0.579 - 0.705	< 0.001
RNFL+GCL+IPL _6.8	0.674	0.613 - 0.735	< 0.001	0.668	0.607 - 0.73	< 0.001
RNFL+GCL+IPL _7.1	0.627	0.564 - 0.691	< 0.001	0.654	0.592 - 0.717	< 0.001
RNFL+GCL+IPL _7.2	0.67	0.608 - 0.731	< 0.001	0.664	0.603 - 0.726	< 0.001
RNFL+GCL+IPL_7.3	0.692	0.632 - 0.751	< 0.001	0.692	0.633 - 0.752	< 0.001
RNFL+GCL+IPL_7.4	0.689	0.629 - 0.749	< 0.001	0.693	0.633 - 0.752	< 0.001
RNFL+GCL+IPL _7.5	0.693	0.633 - 0.753	< 0.001	0.686	0.626 - 0.747	< 0.001
RNFL+GCL+IPL _7.6	0.692	0.632 - 0.752	< 0.001	0.688	0.627 - 0.748	< 0.001
RNFL+GCL+IPL _7.7	0.697	0.638 - 0.757	< 0.001	0.694	0.634 - 0.753	< 0.001
RNFL+GCL+IPL _7.8	0.743	0.688 - 0.798	< 0.001	0.72	0.663 - 0.778	< 0.001
RNFL+GCL+IPL _8.1	0.626	0.562 - 0.69	< 0.001	0.627	0.563 - 0.691	< 0.001
RNFL+GCL+IPL _8.2	0.652	0.589 - 0.714	< 0.001	0.668	0.606 - 0.729	< 0.001
RNFL+GCL+IPL _8.3	0.686	0.625 - 0.746	< 0.001	0.686	0.626 - 0.747	< 0.001
RNFL+GCL+IPL _8.4	0.697	0.638 - 0.757	< 0.001	0.699	0.64 - 0.759	< 0.001
RNFL+GCL+IPL _8.5	0.709	0.65 - 0.768	< 0.001	0.716	0.658 - 0.774	< 0.001
RNFL+GCL+IPL _8.6	0.724	0.667 - 0.781	< 0.001	0.725	0.668 - 0.782	< 0.001
RNFL+GCL+IPL _8.7	0.739	0.683 - 0.796	< 0.001	0.738	0.682 - 0.794	< 0.001
RNFL+GCL+IPL _8.8	0.726	0.669 - 0.784	< 0.001	0.751	0.696 - 0.806	< 0.001

Table S5. Area Under the Receiver Operating Characteristic Curve (AUROC) value, 95% Confidence Interval (CI) and p-value for each cell of the 8x8 macular grid of the ganglion cell layer+inner plexiform layer (GCLIPL) with the grid tilted at 7° and with the grid horizontalized.

	INCLINED 7°			HORIZONTAL		
	**AUROC**	**CI 95%**	***p*-value**	**AUROC**	**CI 95%**	***p*-value**
GCLIPL_1.1	0.563	0.497 - 0.629	0.064	0.575	0.509 - 0.64	0.029
GCLIPL _1.2	0.588	0.523 - 0.654	0.009	0.61	0.545 - 0.675	0.001
GCLIPL _1.3	0.62	0.555 - 0.684	< 0.001	0.635	0.571 - 0.699	< 0.001
GCLIPL _1.4	0.651	0.588 - 0.714	< 0.001	0.658	0.595 - 0.721	< 0.001
GCLIPL _1.5	0.628	0.564 - 0.692	< 0.001	0.637	0.573 - 0.7	< 0.001
GCLIPL _1.6	0.557	0.491 - 0.623	0.095	0.569	0.503 - 0.635	0.044
GCLIPL _1.7	0.473	0.406 - 0.54	0.431	0.48	0.413 - 0.547	0.559
GCLIPL _1.8	0.494	0.427 - 0.561	0.858	0.501	0.434 - 0.568	0.98
GCLIPL _2.1	0.641	0.577 - 0.704	< 0.001	0.659	0.597 - 0.721	< 0.001
GCLIPL _2.2	0.704	0.644 - 0.763	< 0.001	0.691	0.631 - 0.752	< 0.001
GCLIPL _2.3	0.72	0.662 - 0.778	< 0.001	0.732	0.675 - 0.789	< 0.001
GCLIPL _2.4	0.717	0.658 - 0.775	< 0.001	0.709	0.65 - 0.768	< 0.001
GCLIPL _2.5	0.726	0.668 - 0.785	< 0.001	0.726	0.667 - 0.785	< 0.001
GCLIPL _2.6	0.691	0.63 - 0.751	< 0.001	0.686	0.625 - 0.747	< 0.001
GCLIPL _2.7	0.609	0.545 - 0.674	0.001	0.615	0.55 - 0.68	0.001
GCLIPL _2.8	0.47	0.403 - 0.537	0.374	0.42	0.354 - 0.486	0.019
GCLIPL _3.1	0.698	0.638 - 0.758	< 0.001	0.706	0.647 - 0.765	< 0.001
GCLIPL _3.2	0.738	0.681 - 0.795	< 0.001	0.745	0.689 - 0.802	< 0.001
GCLIPL _3.3	0.75	0.693 - 0.806	< 0.001	0.749	0.693 - 0.806	< 0.001
GCLIPL _3.4	0.752	0.696 - 0.808	< 0.001	0.746	0.69 - 0.803	< 0.001
GCLIPL _3.5	0.732	0.674 - 0.79	< 0.001	0.737	0.68 - 0.794	< 0.001
GCLIPL _3.6	0.686	0.626 - 0.747	< 0.001	0.688	0.628 - 0.749	< 0.001
GCLIPL _3.7	0.68	0.619 - 0.741	< 0.001	0.677	0.615 - 0.738	< 0.001
GCLIPL _3.8	0.619	0.555 - 0.683	< 0.001	0.632	0.568 - 0.696	< 0.001
GCLIPL _4.1	0.737	0.68 - 0.793	< 0.001	0.729	0.672 - 0.787	< 0.001
GCLIPL _4.2	0.774	0.72 - 0.828	< 0.001	0.763	0.708 - 0.818	< 0.001
GCLIPL _4.3	0.736	0.679 - 0.792	< 0.001	0.73	0.673 - 0.788	< 0.001
GCLIPL _4.4	0.663	0.601 - 0.725	< 0.001	0.665	0.603 - 0.727	< 0.001
GCLIPL _4.5	0.612	0.548 - 0.676	0.001	0.618	0.554 - 0.682	0.001
GCLIPL _4.6	0.66	0.598 - 0.723	< 0.001	0.658	0.595 - 0.72	< 0.001
GCLIPL _4.7	0.661	0.599 - 0.723	< 0.001	0.66	0.598 - 0.722	< 0.001
GCLIPL _4.8	0.627	0.564 - 0.691	< 0.001	0.642	0.579 - 0.705	< 0.001
GCLIPL _5.1	0.705	0.646 - 0.764	< 0.001	0.698	0.639 - 0.758	< 0.001
GCLIPL _5.2	0.729	0.671 - 0.786	< 0.001	0.707	0.648 - 0.766	< 0.001
GCLIPL _5.3	0.711	0.652 - 0.769	< 0.001	0.702	0.643 - 0.762	< 0.001
GCLIPL _5.4	0.645	0.582 - 0.708	< 0.001	0.643	0.58 - 0.706	< 0.001

GCLIPL _5.5	0.636	0.572 - 0.699	< 0.001	0.622	0.558 - 0.686	< 0.001
GCLIPL _5.6	0.657	0.595 - 0.72	< 0.001	0.651	0.588 - 0.714	< 0.001
GCLIPL _5.7	0.65	0.587 - 0.713	< 0.001	0.64	0.576 - 0.703	< 0.001
GCLIPL _5.8	0.607	0.542 - 0.671	0.002	0.621	0.557 - 0.684	< 0.001
GCLIPL _6.1	0.687	0.627 - 0.747	< 0.001	0.678	0.617 - 0.739	< 0.001
GCLIPL _6.2	0.713	0.655 - 0.771	< 0.001	0.717	0.659 - 0.775	< 0.001
GCLIPL _6.3	0.72	0.662 - 0.779	< 0.001	0.711	0.652 - 0.77	< 0.001
GCLIPL _6.4	0.687	0.626 - 0.747	< 0.001	0.683	0.622 - 0.744	< 0.001
GCLIPL _6.5	0.669	0.607 - 0.73	< 0.001	0.669	0.607 - 0.731	< 0.001
GCLIPL _6.6	0.661	0.599 - 0.723	< 0.001	0.654	0.592 - 0.717	< 0.001
GCLIPL _6.7	0.649	0.587 - 0.712	< 0.001	0.633	0.569 - 0.696	< 0.001
GCLIPL _6.8	0.578	0.512 - 0.643	0.023	0.58	0.514 - 0.645	0.019
GCLIPL _7.1	0.637	0.574 - 0.7	< 0.001	0.652	0.589 - 0.715	< 0.001
GCLIPL _7.2	0.685	0.625 - 0.745	< 0.001	0.673	0.612 - 0.734	< 0.001
GCLIPL _7.3	0.702	0.643 - 0.762	< 0.001	0.697	0.638 - 0.757	< 0.001
GCLIPL _7.4	0.685	0.625 - 0.745	< 0.001	0.682	0.621 - 0.742	< 0.001
GCLIPL _7.5	0.677	0.616 - 0.738	< 0.001	0.67	0.608 - 0.732	< 0.001
GCLIPL _7.6	0.655	0.592 - 0.717	< 0.001	0.653	0.591 - 0.716	< 0.001
GCLIPL _7.7	0.649	0.586 - 0.712	< 0.001	0.614	0.549 - 0.678	0.001
GCLIPL _7.8	0.508	0.441 - 0.575	0.82	0.487	0.42 - 0.553	0.697
GCLIPL _8.1	0.618	0.553 - 0.682	0.001	0.595	0.53 - 0.66	0.005
GCLIPL _8.2	0.617	0.552 - 0.681	0.001	0.629	0.565 - 0.693	< 0.001
GCLIPL _8.3	0.645	0.582 - 0.707	< 0.001	0.639	0.576 - 0.701	< 0.001
GCLIPL _8.4	0.649	0.586 - 0.711	< 0.001	0.645	0.583 - 0.708	< 0.001
GCLIPL _8.5	0.671	0.61 - 0.732	< 0.001	0.663	0.601 - 0.725	< 0.001
GCLIPL _8.6	0.624	0.56 - 0.688	< 0.001	0.625	0.562 - 0.689	< 0.001
GCLIPL _8.7	0.565	0.498 - 0.631	0.057	0.568	0.502 - 0.635	0.045
GCLIPL _8.8	0.468	0.401 - 0.536	0.35	0.426	0.359 - 0.492	0.029

Table S6. Area Under the Receiver Operating Characteristic Curve (AUROC) value, 95% Confidence Interval (CI) and p-value for each cell of the 8x8 macular grid of the outer plexiform layer+outer nuclear layer (OPLONL) with the grid tilted at 7° and with the grid horizontalized.

	INCLINED 7°			HORIZONTAL		
	**AUROC**	**CI 95%**	***p*-value**	**AUROC**	**CI 95%**	***p*-value**
OPLONL_1.1	0.531	0.465 - 0.597	0.363	0.527	0.461 - 0.594	0.423
OPLONL _1.2	0.525	0.459 - 0.592	0.455	0.523	0.457 - 0.59	0.494
OPLONL _1.3	0.537	0.47 - 0.603	0.282	0.527	0.461 - 0.594	0.423
OPLONL _1.4	0.555	0.489 - 0.621	0.104	0.55	0.484 - 0.616	0.146
OPLONL _1.5	0.558	0.492 - 0.624	0.087	0.56	0.494 - 0.626	0.076
OPLONL _1.6	0.557	0.491 - 0.623	0.094	0.566	0.5 - 0.632	0.052
OPLONL _1.7	0.627	0.563 - 0.691	< 0.001	0.622	0.559 - 0.686	< 0.001
OPLONL _1.8	0.601	0.537 - 0.666	0.003	0.6	0.535 - 0.665	0.003
OPLONL _2.1	0.486	0.42 - 0.552	0.679	0.493	0.427 - 0.56	0.842
OPLONL _2.2	0.51	0.443 - 0.576	0.776	0.507	0.44 - 0.573	0.841
OPLONL _2.3	0.529	0.463 - 0.596	0.394	0.529	0.462 - 0.595	0.401
OPLONL _2.4	0.55	0.483 - 0.616	0.144	0.546	0.479 - 0.612	0.181
OPLONL _2.5	0.554	0.487 - 0.62	0.116	0.557	0.491 - 0.624	0.094
OPLONL _2.6	0.529	0.462 - 0.595	0.399	0.529	0.463 - 0.596	0.388
OPLONL _2.7	0.548	0.481 - 0.614	0.163	0.556	0.49 - 0.622	0.099
OPLONL _2.8	0.611	0.547 - 0.676	0.001	0.605	0.54 - 0.669	0.002
OPLONL _3.1	0.502	0.435 - 0.568	0.964	0.492	0.425 - 0.558	0.804
OPLONL _3.2	0.528	0.462 - 0.595	0.409	0.533	0.467 - 0.6	0.328
OPLONL _3.3	0.581	0.515 - 0.646	0.018	0.583	0.517 - 0.649	0.015
OPLONL _3.4	0.612	0.548 - 0.677	0.001	0.609	0.544 - 0.674	0.001
OPLONL _3.5	0.629	0.564 - 0.693	< 0.001	0.628	0.564 - 0.693	< 0.001
OPLONL _3.6	0.583	0.518 - 0.649	0.014	0.586	0.52 - 0.652	0.011
OPLONL _3.7	0.545	0.479 - 0.611	0.184	0.55	0.484 - 0.616	0.141
OPLONL _3.8	0.536	0.47 - 0.602	0.287	0.558	0.493 - 0.624	0.086
OPLONL _4.1	0.507	0.441 - 0.574	0.829	0.524	0.457 - 0.59	0.489
OPLONL _4.2	0.573	0.507 - 0.639	0.033	0.566	0.5 - 0.632	0.052
OPLONL _4.3	0.625	0.561 - 0.689	< 0.001	0.615	0.55 - 0.679	0.001
OPLONL _4.4	0.601	0.537 - 0.666	0.003	0.597	0.532 - 0.661	0.005
OPLONL _4.5	0.567	0.501 - 0.632	0.05	0.572	0.506 - 0.637	0.035
OPLONL _4.6	0.579	0.513 - 0.645	0.02	0.578	0.512 - 0.644	0.022
OPLONL _4.7	0.515	0.449 - 0.582	0.655	0.521	0.454 - 0.587	0.541
OPLONL _4.8	0.515	0.449 - 0.581	0.661	0.511	0.445 - 0.577	0.751
OPLONL _5.1	0.52	0.453 - 0.587	0.559	0.51	0.444 - 0.577	0.764
OPLONL _5.2	0.55	0.483 - 0.616	0.144	0.546	0.48 - 0.613	0.174
OPLONL _5.3	0.589	0.523 - 0.654	0.009	0.586	0.52 - 0.651	0.012
OPLONL _5.4	0.566	0.5 - 0.632	0.053	0.569	0.503 - 0.635	0.042

OPLONL _5.5	0.54	0.474 - 0.606	0.239	0.544	0.478 - 0.611	0.191
OPLONL _5.6	0.571	0.505 - 0.636	0.038	0.58	0.514 - 0.645	0.019
OPLONL _5.7	0.528	0.462 - 0.595	0.405	0.529	0.463 - 0.595	0.396
OPLONL _5.8	0.501	0.434 - 0.568	0.975	0.5	0.433 - 0.566	0.99
OPLONL _6.1	0.502	0.436 - 0.569	0.95	0.489	0.423 - 0.556	0.753
OPLONL _6.2	0.514	0.447 - 0.581	0.682	0.505	0.438 - 0.572	0.886
OPLONL _6.3	0.524	0.457 - 0.59	0.483	0.521	0.454 - 0.587	0.539
OPLONL _6.4	0.56	0.494 - 0.626	0.079	0.556	0.49 - 0.622	0.1
OPLONL _6.5	0.545	0.479 - 0.612	0.183	0.55	0.483 - 0.616	0.145
OPLONL _6.6	0.537	0.47 - 0.603	0.281	0.531	0.464 - 0.597	0.365
OPLONL _6.7	0.522	0.455 - 0.589	0.515	0.514	0.447 - 0.58	0.684
OPLONL _6.8	0.53	0.463 - 0.596	0.384	0.502	0.436 - 0.569	0.946
OPLONL _7.1	0.489	0.423 - 0.556	0.756	0.483	0.416 - 0.549	0.611
OPLONL _7.2	0.479	0.413 - 0.546	0.542	0.482	0.416 - 0.549	0.605
OPLONL _7.3	0.503	0.436 - 0.57	0.933	0.498	0.431 - 0.564	0.943
OPLONL _7.4	0.497	0.43 - 0.564	0.927	0.501	0.434 - 0.568	0.978
OPLONL _7.5	0.518	0.451 - 0.585	0.592	0.52	0.453 - 0.587	0.555
OPLONL _7.6	0.525	0.459 - 0.592	0.454	0.526	0.46 - 0.593	0.437
OPLONL _7.7	0.531	0.465 - 0.598	0.355	0.524	0.457 - 0.591	0.478
OPLONL _7.8	0.563	0.497 - 0.629	0.065	0.573	0.507 - 0.639	0.031
OPLONL _8.1	0.472	0.406 - 0.538	0.409	0.463	0.397 - 0.529	0.278
OPLONL _8.2	0.464	0.398 - 0.531	0.295	0.481	0.414 - 0.547	0.569
OPLONL _8.3	0.476	0.41 - 0.543	0.49	0.492	0.426 - 0.559	0.82
OPLONL _8.4	0.49	0.423 - 0.557	0.769	0.495	0.429 - 0.562	0.895
OPLONL _8.5	0.506	0.439 - 0.573	0.857	0.511	0.444 - 0.577	0.758
OPLONL _8.6	0.519	0.452 - 0.586	0.583	0.533	0.467 - 0.6	0.329
OPLONL _8.7	0.562	0.496 - 0.629	0.067	0.562	0.496 - 0.628	0.068
OPLONL _8.8	0.557	0.49 - 0.623	0.096	0.569	0.503 - 0.635	0.043

Table S7. Area Under the Receiver Operating Characteristic Curve (AUROC) value, 95% Confidence Interval (CI) and p-value for each cell of the 8x8 macular grid of the inner retinal layers (INL) with the grid tilted at 7° and with the grid horizontalized.

	INCLINED 7°			HORIZONTAL		
	**AUROC**	**CI 95%**	***p*-value**	**AUROC**	**CI 95%**	***p*-value**
IRL_1.1	0.611	0.546 - 0.675	0.001	0.612	0.547 - 0.676	0.001
IRL _1.2	0.644	0.582 - 0.707	< 0.001	0.626	0.563 - 0.69	< 0.001
IRL _1.3	0.675	0.614 - 0.736	< 0.001	0.678	0.617 - 0.739	< 0.001
IRL _1.4	0.684	0.624 - 0.745	< 0.001	0.681	0.621 - 0.742	< 0.001
IRL _1.5	0.691	0.63 - 0.752	< 0.001	0.688	0.627 - 0.748	< 0.001
IRL 1.6	0.718	0.66 - 0.776	< 0.001	0.701	0.641 - 0.76	< 0.001
IRL _1.7	0.737	0.681 - 0.794	< 0.001	0.732	0.675 - 0.789	< 0.001
IRL _1.8	0.726	0.668 - 0.783	< 0.001	0.721	0.664 - 0.779	< 0.001
IRL _2.1	0.614	0.55 - 0.679	0.001	0.617	0.553 - 0.681	0.001
IRL _2.2	0.661	0.599 - 0.723	< 0.001	0.65	0.587 - 0.712	< 0.001
IRL _2.3	0.672	0.61 - 0.733	< 0.001	0.672	0.61 - 0.733	< 0.001
IRL _2.4	0.662	0.6 - 0.724	< 0.001	0.658	0.595 - 0.72	< 0.001
IRL _2.5	0.684	0.623 - 0.745	< 0.001	0.675	0.613 - 0.736	< 0.001
IRL _2.6	0.693	0.633 - 0.753	< 0.001	0.696	0.636 - 0.757	< 0.001
IRL 2.7	0.706	0.647 - 0.766	< 0.001	0.716	0.658 - 0.775	< 0.001
IRL 2.8	0.769	0.715 - 0.822	< 0.001	0.756	0.701 - 0.811	< 0.001
IRL _3.1	0.616	0.552 - 0.681	0.001	0.608	0.543 - 0.672	0.002
IRL _3.2	0.645	0.582 - 0.708	< 0.001	0.637	0.573 - 0.7	< 0.001
IRL _3.3	0.656	0.594 - 0.719	< 0.001	0.651	0.589 - 0.714	< 0.001
IRL 3.4	0.675	0.613 - 0.736	< 0.001	0.666	0.604 - 0.728	< 0.001
IRL _3.5	0.66	0.598 - 0.722	< 0.001	0.667	0.605 - 0.729	< 0.001
IRL 3.6	0.641	0.578 - 0.704	< 0.001	0.647	0.584 - 0.71	< 0.001
IRL _3.7	0.664	0.602 - 0.726	< 0.001	0.666	0.605 - 0.728	< 0.001
IRL _3.8	0.691	0.631 - 0.751	< 0.001	0.716	0.657 - 0.774	< 0.001
IRL _4.1	0.593	0.528 - 0.658	0.006	0.581	0.515 - 0.646	0.018
IRL _4.2	0.627	0.564 - 0.691	< 0.001	0.613	0.549 - 0.677	0.001
IRL _4.3	0.629	0.566 - 0.693	< 0.001	0.614	0.55 - 0.679	0.001
IRL _4.4	0.552	0.486 - 0.618	0.124	0.546	0.48 - 0.612	0.174
IRL _4.5	0.527	0.461 - 0.593	0.43	0.527	0.46 - 0.593	0.432
IRL _4.6	0.571	0.505 - 0.636	0.038	0.574	0.508 - 0.639	0.03
IRL _4.7	0.594	0.529 - 0.659	0.006	0.611	0.546 - 0.675	0.001
IRL _4.8	0.607	0.543 - 0.672	0.002	0.637	0.573 - 0.7	< 0.001
IRL _5.1	0.563	0.497 - 0.629	0.066	0.568	0.502 - 0.633	0.047
IRL _5.2	0.579	0.514 - 0.645	0.02	0.589	0.524 - 0.654	0.009
IRL _5.3	0.593	0.528 - 0.658	0.006	0.597	0.532 - 0.662	0.004
IRL _5.4	0.542	0.476 - 0.608	0.218	0.541	0.475 - 0.607	0.225

IRL _5.5	0.545	0.479 - 0.611	0.189	0.531	0.465 - 0.598	0.356
IRL _5.6	0.567	0.501 - 0.632	0.05	0.56	0.494 - 0.626	0.079
IRL _5.7	0.578	0.513 - 0.644	0.021	0.575	0.509 - 0.64	0.028
IRL _5.8	0.603	0.538 - 0.667	0.003	0.59	0.525 - 0.655	0.008
IRL 6.1	0.564	0.499 - 0.63	0.059	0.583	0.518 - 0.649	0.014
IRL _6.2	0.606	0.542 - 0.67	0.002	0.607	0.542 - 0.671	0.002
IRL _6.3	0.618	0.554 - 0.682	0.001	0.616	0.552 - 0.679	0.001
IRL 6.4	0.616	0.552 - 0.68	0.001	0.617	0.553 - 0.681	0.001
IRL _6.5	0.608	0.543 - 0.672	0.002	0.6	0.535 - 0.665	0.003
IRL _6.6	0.616	0.552 - 0.68	0.001	0.603	0.538 - 0.667	0.003
IRL _6.7	0.627	0.563 - 0.691	< 0.001	0.606	0.542 - 0.671	0.002
IRL 6.8	0.644	0.581 - 0.706	< 0.001	0.634	0.571 - 0.698	< 0.001
IRL 7.1	0.576	0.511 - 0.642	0.025	0.59	0.525 - 0.655	0.008
IRL _7.2	0.61	0.545 - 0.674	0.001	0.612	0.548 - 0.677	0.001
IRL _7.3	0.627	0.563 - 0.69	< 0.001	0.63	0.566 - 0.693	< 0.001
IRL 7.4	0.628	0.565 - 0.691	< 0.001	0.63	0.567 - 0.694	< 0.001
IRL _7.5	0.636	0.573 - 0.699	< 0.001	0.635	0.571 - 0.698	< 0.001
IRL 7.6	0.641	0.579 - 0.704	< 0.001	0.638	0.575 - 0.701	< 0.001
IRL 7.7	0.668	0.606 - 0.729	< 0.001	0.662	0.6 - 0.724	< 0.001
IRL _7.8	0.709	0.65 - 0.767	< 0.001	0.685	0.625 - 0.745	< 0.001
IRL _8.1	0.596	0.531 - 0.66	0.005	0.604	0.54 - 0.669	0.002
IRL _8.2	0.621	0.557 - 0.685	< 0.001	0.63	0.567 - 0.694	< 0.001
IRL 8.3	0.649	0.586 - 0.711	< 0.001	0.648	0.586 - 0.711	< 0.001
IRL 8.4	0.656	0.594 - 0.718	< 0.001	0.662	0.6 - 0.724	< 0.001
IRL _8.5	0.672	0.611 - 0.733	< 0.001	0.681	0.621 - 0.742	< 0.001
IRL _8.6	0.699	0.639 - 0.758	< 0.001	0.698	0.639 - 0.757	< 0.001
IRL _8.7	0.71	0.651 - 0.768	< 0.001	0.712	0.654 - 0.77	< 0.001
IRL 8.8	0.709	0.651 - 0.767	< 0.001	0.72	0.662 - 0.778	< 0.001

Table S8. Area Under the Receiver Operating Characteristic Curve (AUROC) value, 95% Confidence Interval (CI) and p-value for each cell of the 8x8 macular grid of the outer retinal layer (ORL) with the grid tilted at 7° and with the grid horizontalized.

	INCLINED 7°			HORIZONTAL		
	AUROC	CI 95%	*p*-value	AUROC	CI 95%	*p*-value
ORL_1.1	0.555	0.488 - 0.621	0.109	0.539	0.473 - 0.606	0.251
ORL _1.2	0.572	0.507 - 0.638	0.034	0.558	0.492 - 0.625	0.086
ORL _1.3	0.561	0.495 - 0.628	0.071	0.539	0.473 - 0.606	0.248
ORL _1.4	0.572	0.506 - 0.638	0.036	0.568	0.502 - 0.635	0.045
ORL _1.5	0.555	0.489 - 0.622	0.104	0.568	0.502 - 0.634	0.047
ORL _1.6	0.587	0.521 - 0.652	0.011	0.585	0.52 - 0.651	0.012
ORL _1.7	0.586	0.52 - 0.651	0.012	0.587	0.522 - 0.653	0.01
ORL _1.8	0.545	0.479 - 0.611	0.183	0.581	0.516 - 0.646	0.018
ORL _2.1	0.535	0.468 - 0.601	0.31	0.534	0.468 - 0.6	0.318
ORL _2.2	0.543	0.477 - 0.609	0.21	0.534	0.468 - 0.6	0.317
ORL _2.3	0.533	0.467 - 0.599	0.333	0.519	0.452 - 0.585	0.585
ORL _2.4	0.527	0.461 - 0.593	0.428	0.523	0.457 - 0.59	0.495
ORL _2.5	0.552	0.486 - 0.618	0.125	0.55	0.484 - 0.616	0.14
ORL _2.6	0.57	0.504 - 0.635	0.041	0.565	0.499 - 0.63	0.058
ORL _2.7	0.579	0.514 - 0.645	0.02	0.604	0.539 - 0.668	0.002
ORL _2.8	0.56	0.495 - 0.626	0.076	0.57	0.504 - 0.636	0.039
ORL _3.1	0.543	0.477 - 0.61	0.202	0.548	0.482 - 0.614	0.16
ORL _3.2	0.529	0.463 - 0.596	0.391	0.526	0.459 - 0.592	0.448
ORL _3.3	0.517	0.451 - 0.584	0.611	0.51	0.443 - 0.576	0.78
ORL _3.4	0.508	0.442 - 0.574	0.814	0.51	0.443 - 0.576	0.774
ORL _3.5	0.539	0.472 - 0.605	0.258	0.522	0.455 - 0.588	0.526
ORL _3.6	0.542	0.476 - 0.608	0.221	0.54	0.474 - 0.606	0.237
ORL _3.7	0.536	0.469 - 0.602	0.293	0.567	0.501 - 0.632	0.051
ORL _3.8	0.519	0.453 - 0.586	0.575	0.566	0.5 - 0.631	0.054
ORL _4.1	0.546	0.48 - 0.613	0.173	0.541	0.475 - 0.608	0.224
ORL _4.2	0.514	0.447 - 0.58	0.691	0.506	0.44 - 0.572	0.856
ORL 4.3	0.478	0.411 - 0.544	0.509	0.479	0.413 - 0.546	0.543
ORL _4.4	0.439	0.373 - 0.504	0.071	0.442	0.377 - 0.508	0.091
ORL _4.5	0.442	0.376 - 0.508	0.086	0.45	0.384 - 0.516	0.141
ORL _4.6	0.487	0.421 - 0.554	0.708	0.507	0.44 - 0.574	0.836
ORL _4.7	0.494	0.428 - 0.561	0.867	0.517	0.45 - 0.583	0.625
ORL_ 4.8	0.516	0.45 - 0.583	0.633	0.515	0.448 - 0.582	0.657
ORL _5.1	0.54	0.474 - 0.607	0.235	0.533	0.467 - 0.599	0.333
ORL_5.2	0.52	0.454 - 0.587	0.553	0.517	0.451 - 0.583	0.616
ORL _5.3	0.49	0.423 - 0.556	0.76	0.488	0.422 - 0.555	0.734
ORL _5.4	0.453	0.387 - 0.519	0.17	0.454	0.388 - 0.52	0.173

ORL _5.5	0.44	0.375 - 0.506	0.08	0.44	0.374 - 0.506	0.077
ORL _5.6	0.469	0.403 - 0.536	0.366	0.456	0.389 - 0.522	0.191
ORL _5.7	0.498	0.431 - 0.564	0.943	0.501	0.435 - 0.568	0.967
ORL _5.8	0.492	0.425 - 0.559	0.813	0.515	0.448 - 0.583	0.65
ORL _6.1	0.525	0.459 - 0.591	0.463	0.506	0.439 - 0.572	0.861
ORL _6.2	0.493	0.427 - 0.56	0.844	0.482	0.416 - 0.548	0.597
ORL _6.3	0.503	0.436 - 0.569	0.938	0.502	0.435 - 0.568	0.959
ORL _6.4	0.503	0.437 - 0.569	0.93	0.508	0.442 - 0.575	0.804
ORL _6.5	0.514	0.447 - 0.58	0.686	0.511	0.445 - 0.578	0.744
ORL _6.6	0.532	0.466 - 0.598	0.35	0.521	0.455 - 0.588	0.532
ORL _6.7	0.493	0.426 - 0.56	0.832	0.494	0.427 - 0.561	0.858
ORL _6.8	0.526	0.459 - 0.592	0.453	0.5	0.433 - 0.567	0.991
ORL _7.1	0.496	0.429 - 0.563	0.905	0.51	0.444 - 0.577	0.762
ORL _7.2	0.514	0.447 - 0.58	0.692	0.516	0.449 - 0.582	0.644
ORL _7.3	0.515	0.448 - 0.581	0.666	0.518	0.452 - 0.585	0.594
ORL _7.4	0.509	0.442 - 0.575	0.798	0.505	0.438 - 0.571	0.894
ORL _7.5	0.508	0.442 - 0.575	0.812	0.508	0.442 - 0.575	0.808
ORL _7.6	0.549	0.483 - 0.615	0.148	0.53	0.463 - 0.596	0.384
ORL _7.7	0.524	0.457 - 0.59	0.483	0.522	0.456 - 0.589	0.512
ORL _7.8	0.523	0.457 - 0.59	0.497	0.538	0.472 - 0.604	0.264
ORL _8.1	0.502	0.435 - 0.569	0.952	0.503	0.437 - 0.57	0.926
ORL _8.2	0.495	0.428 - 0.561	0.879	0.497	0.43 - 0.563	0.919
ORL _8.3	0.484	0.418 - 0.551	0.643	0.473	0.407 - 0.539	0.428
ORL _8.4	0.502	0.435 - 0.568	0.955	0.507	0.44 - 0.573	0.845
ORL _8.5	0.516	0.449 - 0.582	0.644	0.541	0.475 - 0.607	0.227
ORL _8.6	0.551	0.485 - 0.616	0.137	0.544	0.478 - 0.61	0.198
ORL _8.7	0.532	0.466 - 0.598	0.345	0.559	0.493 - 0.625	0.083
ORL _8.8	0.543	0.477 - 0.609	0.207	0.563	0.497 - 0.629	0.065

Table S9. AUROC and comparison of mean and weighted indices for the 8x8 grid of the Posterior Pole (PP) using Spectralis SD-OCT in the thickness of the complete retina.

Complete retina	AUROC	CI 95%	p-value
**Horizontilized**			
Mean index	0.7432	0.688 - 0.798	< 0.001
Weighted index	0.7409	0.686 - 0.796	< 0.001
Mean vs. Weighted	0.0023	-0.01 - 0.015	0.712
**Inclined**			
Mean index	0.7518	0.698 - 0.806	< 0.001
Weighted index	0.7523	0.698 - 0.806	< 0.001
Mean vs. Weighted	-0.0005	-0.001 - 0	0.223

Table S10. AUROC and comparison of mean and weighted indices for the 8x8 grid of the Posterior Pole (PP) using Spectralis SD-OCT in the thickness of the RNFL..

RNFL	AUROC	CI 95%	p-value
**Horizontilized**			
Mean index	0.7937	0.744 - 0.843	< 0.001
Weighted index	0.7941	0.744 - 0.844	< 0.001
Mean vs. Weighted	-0.0004	-0.002 - 0.001	0.489
**Inclined**			
Mean index	0.7959	0.746 - 0.845	< 0.001
Weighted index	0.7961	0.747 - 0.846	< 0.001
Mean vs. Weighted	-0.0002	-0.001 - 0	0.443

Table S2. AUROC and comparison of mean and weighted indices for the 8x8 grid of the Posterior Pole (PP) using Spectralis SD-OCT in the thickness of the GCL.

.

GCL	AUROC	CI 95%	p-value
**Horizontilized**			
Mean index	0.7813	0.729 - 0.834	< 0.001
Weighted index	0.7814	0.729 - 0.834	< 0.001
Mean vs. Weighted	-0.0001	-0.001 - 0.001	0.612
**Inclined**			
Mean index	0.7841	0.732 - 0.836	< 0.001
Weighted index	0.784	0.732 - 0.836	< 0.001
Mean vs. Weighted	0.0001	-0.001 - 0.001	0.799

Table S12. AUROC and comparison of mean and weighted indices for the 8x8 grid of the Posterior Pole (PP) using Spectralis SD-OCT in the thickness of the ganglion cell complex.

Ganglion cell complex	AUROC	CI 95%	*p*-value
**Horizontilized**			
Mean index	0.7842	0.733 - 0.835	< 0.001
Weighted index	0.7844	0.734 - 0.835	< 0.001
Mean vs. Weighted	-0.0002	-0.001 - 0.001	0.701
**Inclined**			
Mean index	0.7846	0.734 - 0.835	< 0.001
Weighted index	0.785	0.734 - 0.836	< 0.001
Mean vs. Weighted	-0.0004	-0.001 - 0	0.266

Table S13. AUROC and comparison of mean and weighted indices for the 8x8 grid of the Posterior Pole (PP) using Spectralis SD-OCT in the thickness of GCLIPL.

**GCLIPL**		**AUROC**	**CI 95%**	***p*-value**
**Horizontilized**				
Mean index		0.7687	0.715 - 0.823	< 0.001
Weighted index		0.769	0.715 - 0.823	< 0.001
	Mean vs. Weighted	-0.0003	-0.001 - 0	0.354
**Inclined**				
Mean index		0.7701	0.717 - 0.824	< 0.001
Weighted index		0.7703	0.717 - 0.824	< 0.001
	Mean vs. Weighted	-0.0002	-0.001 - 0	0.403

Table S14. AUROC and comparison of mean and weighted indices for the 8x8 grid of the Posterior Pole (PP) using Spectralis SD-OCT in the thickness of the IRL.

**IRL**		**AUROC**	**CI 95%**	***p*-value**
**Horizontilized**				
Mean index		0.7513	0.697 - 0.806	< 0.001
Weighted index		0.7505	0.696 - 0.805	< 0.001
	Mean vs. Weighted	0.0008	-0.008 - 0.01	0.861
**Inclined**				
Mean index		0.7551	0.701 - 0.809	< 0.001
Weighted index		0.7555	0.702 - 0.809	< 0.001
	Mean vs. Weighted	-0.0004	-0.001 - 0	0.287
